# Understanding community resistance to sexuality education and exploring prospective implementation strategies in Pakistan: A content and network analysis of qualitative data

**DOI:** 10.3389/fpsyg.2022.864465

**Published:** 2022-09-15

**Authors:** Furqan Ahmed, Janina Schumacher, Ghufran Ahmad, Tilman Brand

**Affiliations:** ^1^Department of Prevention and Evaluation, Leibniz Institute of Prevention Research and Epidemiology, Bremen, Germany; ^2^Health Sciences Bremen, University of Bremen, Bremen, Germany; ^3^NUST Business School (NBS), National University of Sciences and Technology (NUST), Islamabad, Pakistan

**Keywords:** sexuality education, community resistance, social norms, adolescent health, network analysis, community readiness

## Abstract

Providing comprehensive sexuality education (CSE) in schools is a work in progress in many countries throughout the world. In some countries, the journey is just beginning; in others, investments in this field have been made for many years. It is and has been difficult in Pakistan to implement and promote reproductive health, women’s empowerment, and CSE. In Pakistan, previous implementation efforts revealed the critical role of community influencers in propagating misleading information about the initiatives, inciting organized community resistance, and provoking backlash. This paper looked at several aspects of community resistance, as well as approaches for overcoming the resistance for increasing community engagement in the implementation of CSE in Islamabad, Pakistan. To analyze community perceptions of CSE implementation in Islamabad, the community readiness assessment (CRA) questionnaire was adapted. Questions and prompts for discussion included leadership, current initiatives, community knowledge, resource availability, community support, and implementation strategies. A total of 35 in-depth interviews were conducted. Data was analyzed and interpreted using qualitative content analysis to explore community perspectives that contribute to resistance around CSE, as well as implementation options. Using inter-code relationship data, network analysis was conducted to provide a graphical representation of the analyzed qualitative data. The study reveals community resistance to CSE being implemented in schools. Misconceptions, a lack of awareness, a lack of priority, and the lack of dedicated resources are just a few of the primary implementation challenges to consider when implementing CSE in practice. Network analysis identified, based on modularity class, five distinct clusters of highly connected nodes/codes: non-governmental organizations (NGOs), misconceptions, resources and policy, strategies and community support, and personal social and health education (PSHE) and current efforts. In conservative environments and when confronted with resistance, innovative marketing and rebranding are critical for priority setting and community engagement, especially when developing curriculum and implementing CSE. Some of the suggested strategies for implementation include community sensitization through strategic awareness campaigns, involving already established infrastructure and NGOs, endorsement by all major stakeholders, particularly decision-makers, and the use of creative digital platforms for better dissemination.

## Introduction

Adolescent sexual and reproductive health (SRH) education and promotion was advocated by the International Conference on Population and Development in 1994 (Fincher, 1994). Unfortunately, due to misconceptions, organized community resistance and implementation obstacles progress has been slow (UNESCO, 2015). According to United Nations Educational, Scientific, and Cultural Organization‘s (UNESCO) 2021 global status report on comprehensive sexuality education (CSE), countries around the world are at various stages of progression (UNESCO, 2021a,b,c). This journey must continually respond to emerging health and well-being challenges, and the unique requirements of children and adolescents. This includes ensuring that CSE is mandated by law and policy with committed funding for expanding coverage (Ahmed et al., 2021; UNESCO, 2021a,b,c). This can only be accomplished by prioritizing content and delivery quality through curricular reforms and investments in teacher training. As many countries continue on their path to CSE, monitoring progress is critical (UNESCO, 2021a,b,c). This includes improving the use of recommended indicators and incorporating diverse viewpoints, such as those of parents, teachers, learners, and community influencers (Chandra-Mouli et al., 2018b; Ahmed et al., 2020a, 2021; UNESCO, 2021a,b,c).

### Challenges during puberty

Participants in a survey conducted in Pakistan identified the specific health issues they face during puberty and also provided indirect insights into how a lack of open discussion about SRH causes them to experience unnecessary shock, pain, and guilt as they navigate natural physiological changes (Kamran et al., 2019). For example, the majority of girls stated that they were shocked when they started menstruation since they had not been informed that this would occur (Kamran et al., 2019). In the absence of formal CSE, adolescents rely on unreliable sources of information to make decisions about their sexuality (Ali et al., 2006; Talpur and Khowaja, 2012; Iqbal et al., 2017). In addition to exposing children to harm, misinformation, mistreatment, and exploitation, adolescents may also develop mental health issues, according to the findings of a Karachi-based study (Ali et al., 2006; Iqbal et al., 2017).

### Marriage, early marriage, and fertility

In recent years, the singulate mean age at marriage (estimate of average years lived before marriage) for women and men has climbed to around 23 and 27 years, respectively (Kamran et al., 2019; National Institute of Population Studies (NIPS) and ICF, 2019). Despite this, 14% of adolescent females and 3% of adolescent males between the ages of 15 and 19 are married. Moreover, between the ages of 15 and 19, around one-fifth of females have begun childbearing. Additionally, adolescent mothers in Pakistan give birth to 44 children for every 1,000 live births. They are three times more likely than older mothers to be anemic and to have a lower pre-pregnancy body mass index. Additionally, they are three times as likely to miscarriage than older moms (Shah et al., 2011). Additionally, their babies have a greater chance of preterm delivery and low birth weight, as well as an increased risk of dying during childbirth (Shah et al., 2011).

### Planned parenthood and contraception

Around 97% of married women between the ages of 15 and 29 in Pakistan have heard of at least one method of contraception (Kamran et al., 2019; National Institute of Population Studies (NIPS) and ICF, 2019). Women’s levels of knowledge increase with age, from almost 91% of those aged 15–19 to nearly 98% of those aged 25–29. However, only 22% of married women between the ages of 15 and 29 use contraception to plan their families. Contraceptive usage among married women between the ages of 15 and 29 rises with age, is higher among those with a higher level of education and income, and varies depending on the region in which they reside. Additionally, 17.9% of married women between the ages of 15 and 19 report unmet contraception needs (Kamran et al., 2019; National Institute of Population Studies (NIPS) and ICF, 2019).

### Adolescent reproductive health rights

A recent cross-sectional survey reveals that adolescents, parents, and caregivers in Lahore, Pakistan, have a poor understanding of adolescents’ SRH rights (Iqbal et al., 2017). Pakistan ranked 151st out of 153 countries in the World Economic Forum’s 2020 report on gender parity (World Economic Forum, 2021). Females in Pakistan have less financial independence and almost no decision-making power compared to men (UNESCO; World Economic Forum, 2021). In addition, there is a high prevalence of child marriage, and there is little acknowledgement that young girls need education on their sexuality and reproductive health rights (Kamran et al., 2019; National Institute of Population Studies (NIPS) and ICF, 2019).

Adolescent boys and girls are typically aware of human rights, but they are more expressive about their rights as adolescents, according to the results of a qualitative study (Kamran et al., 2019). Girls spoke more frequently about their right to education and marriage consent, while boys discussed their right to employment (Kamran et al., 2019). However, just a limited percentage of adolescents listed the right to be free from child labor, harassment, and assault, and none mentioned the right to be safe from domestic abuse (Kamran et al., 2019). It is vital to raise adolescents’ understanding of their rights, the law, and the institutions of justice.

### Gender-based violence, child abuse, and cyberbullying

A 2018 international survey on men and gender equality in Pakistan revealed that over 59% of women had suffered some sort of violence, while about half of men (50%) had perpetrated violence (Kamran et al., 2018). Over 10% of both men and women reported having been abused as children (Kamran et al., 2019; National Institute of Population Studies (NIPS) and ICF, 2019). In addition, the study uncovered a high degree of intergenerational transmission of violence among respondents. The practice of honor killing, marriage as a means of resolving disputes (vani), and bridal exchange (watta satta) are all examples of destructive customs and traditions that exist in Pakistan and are detrimental to women and girls. Moreover, emotional violence is rampant. Financial issues, infertility, a husband assaulting the children, and a wife’s unwillingness to participate in sexual intercourse are the most often claimed reasons for verbal domestic violence. Pakistan, like many other regions, has a problem with the underreporting of physical, sexual, and other types of violence, especially by female victims.

According to Sahil foundation records, child abuse was recorded in 3,445 instances in 2017, 3,832 instances in 2018, 2,846 instances in 2019, and 2,960 incidences during 2020 (Ahmed et al., 2020b). In nearly two-thirds of these cases, the perpetrators were acquaintances or family members of the victims. There is a significant data gap since there are no accessible data registries for monitoring and reporting the number of child abuse incidents in Pakistan. These statistics are derived through monitoring a range of national and regional newspapers. Due to social stigma associated with child abuse, underreporting is also a critical concern (Ahmed et al., 2020b). Strategies like sex offender management and school-based education programs may help prevent such heinous acts, and CSE programs effectively reduce child abuse, teenage pregnancy, and sexually transmitted diseases (Finkelhor, 2009; Kirby et al., 2011).

According to the results of a qualitative study on cyberbullying, the overwhelming majority of respondents said that men are more likely to engage in cyberbullying since they have better access to internet and mobile devices (Kamran et al., 2019). It is likely that girls will be deceived by fake accounts or harassed by ex-boyfriends who threaten to post their personal photographs on social media. If girls do not get sufficient support from their parents, they may be subjected to additional restrictions on their movement and use of mobile phones, physical abuse, expulsion from school, and even forced marriage (Kamran et al., 2019). With such a high percentage of adolescents using social media sites, it is crucial to raise knowledge about these networks and how to use them safely.

### Implementation challenges and socio-ecological model

According to a 2014 report by UNESCO, there are few instances of scaled-up CSE intervention programs. There is a substantial gap in school-based CSE, as well as its exclusion from official educational curriculum, which poses a substantial barrier to progress in these fields (UNESCO; Sokal and Rohlf, 2015). Pakistan provides a challenging environment in which to implement and promote reproductive health, women’s empowerment, and CSE, as is the case in many conservative societies (Shaikh and Ochani, 2018; Chandra-Mouli et al., 2018b). It is a taboo to discuss adolescent SRH and, like in many other countries, there is a widespread belief that sexual education may lead to undesirable behaviors (Svanemyr et al., 2015; Shaikh and Ochani, 2018; Chandra-Mouli et al., 2018b). Moreover, according to UNESCO, “there is less clarity on how to implement (CSE) and scale it up in varied environments,” particularly when community resistance is observed (UNESCO, 2015).

School-based CSE plays a crucial role in reaching out to a wide number of stakeholders while imparting age-appropriate and developmentally relevant information using a systematic, spiral approach that builds on prior content and sexuality concepts (Herat et al., 2018; Montgomery and Knerr, 2021). According to literature, a substantial proportion of Pakistan’s youth favor the implementation of CSE (Ali et al., 2006; Shaikh et al., 2017; Shaikh and Ochani, 2018). Compared to this, support from community influencers, decision-makers, and gatekeepers is notably low, and a culture of silence around CSE is often seen (Chandra-Mouli et al., 2018b). While community engagement and the development of strategies to counter widespread resistance to CSE have been emphasized, such interventions are rare in Pakistan (Svanemyr et al., 2015; Chandra-Mouli et al., 2018a, 2018b; Ahmed et al., 2021). Two non-governmental organizations (NGOs), Aahung and Rutgers, sought to address the challenge of resistance in school-based and out-of-school adolescent SRH interventions in Pakistan by using a participatory approach to design life skills-based curriculum (Chandra-Mouli et al., 2018b; Rutgers, n.d.). They were successful in drawing attention to the critical role of community influencers and gatekeepers in disseminating disinformation about the projects, inciting organized community resistance, and generating an outpouring of resistance (Chandra-Mouli et al., 2018b). Aahung also conducted a mapping exercise to identify the most influential decision-makers in the lives of adolescents. This study suggested many levels of influence within an adolescent’s context, which must be recognized in order to sensitize and engage influencers in order to approach adolescents with CSE effectively. This includes political and religious authorities (societal tier), community leaders (community tier), school administrators and teachers (organizational tier), and parents and peers (interpersonal tier; Chandra-Mouli et al., 2018b). These influencers and gatekeepers have an impact on adolescent access to CSE and reproductive health.

### Study setting: Islamabad

Our research was conducted in Islamabad, capital city of Pakistan. According to the 2017 census, Islamabad has a population of over 2 million with 0.3 million households (Pakistan Bureau of Statistics, 2017). The city contains 367 primary (grades 1–5), 162 middle (grades 6–8), 250 high (grades 9 and 10), and 75 higher secondary (grades 11 and 12) schools (Ministry of Education, 2009). Literacy rate is 88%, the highest in the country (ICT Administration, n.d.). Literature reveals that Islamabad and Rawalpindi (adjoining cities) provide a similar picture in terms of adolescent reproductive health concerns addressed in previous sections. A survey conducted in Rawalpindi revealed that 48.6% of females only have enough understanding about puberty and menstruation (Mansoor et al., 2021). In addition, urban women were almost four times more likely to have appropriate understanding of puberty and menstruation, whereas working women were nearly 16 times more likely to have adequate understanding. Another study conducted in Islamabad and Rawalpindi on the perceptions of parents and teachers about CSE revealed that 76.1 percent of parents and 64.4 percent of teachers supported the implementation of age-appropriate CSE in schools (Nadeem et al., 2021). But the majority of teachers and families considered that CSE was incompatible with Islamic values and culture. The majority of respondents favored the prevention of bullying and sexual assault, but subjects such as birth control received the least support. Nearly half of the parents said that they had never had a conversation with their children about their sexual health with their children. Therefore, this paper investigates several aspects of community resistance and identifies strategies for community engagement and resistance management for successful CSE implementation in schools in Islamabad.

## Materials and methods

### Research team

FA (Male) interviewed the respondents online. FA and TB had prior expertise in collecting and analyzing qualitative research data (Siddiqi et al., 2016; Bradby et al., 2020). At the time of data collection, FA was a doctoral fellow at the Leibniz Institute of Prevention Research and Epidemiology (BIPS).

### Data collection

Community readiness is a critical factor for the successful implementation of community-based health programs (Edwards et al., 2000; Stith et al., 2006; Oetting et al., 2014). The concept of community readiness in preventive health programs is based on increasing this readiness to have better participation and inclusion in health interventions (Stith et al., 2006). Several tools to assess community readiness have been developed, but the community readiness model (CRM) is the one that has widely been used in health promotion, suicide prevention, HIV/AIDS prevention, and programs aiming to improve physical activity uptake (Edwards et al., 2000; Stith et al., 2006; Peercy et al., 2010; Oetting et al., 2014; Gansefort et al., 2018; Ahmed et al., 2021). The community readiness assessment (CRA) questionnaire was modified to assess perceptions on CSE implementation (Ahmed et al., 2021). Themes such as leadership, current efforts, community knowledge, resource availability, community support, and implementation strategies were among the questions for discussion. The semi-structured interview questionnaire included both open-ended and close-ended rating questions that were written down while writing field notes. The interview questionnaire is provided in Supplementary File as Questionnaire 1. The data was collected and recorded online from April to July 2020. This study received ethics approval from the Pakistan Health Research Council, and all respondents gave their informed consent verbally and in writing through email prior to the interviews.

### Key respondents and recruitment

According to Mouli et al. (Svanemyr et al., 2015; Chandra-Mouli et al., 2018b), there are many levels of the community that impact adolescents’ access to sexuality education in Pakistan. These levels, which correspond to the ecological framework, are divided into five categories: society, community, organizational, interpersonal, and individual (Chandra-Mouli et al., 2018b). Except for the individual level, we classified key respondents from Islamabad for each level because our focus was on gatekeepers and influencers.

Respondents were recruited in each level based on existing research about stakeholders who play an important role in influencing choices about the implementation of health interventions, particularly SRH (Svanemyr et al., 2015; Chandra-Mouli et al., 2018b; Ahmed et al., 2021). Potential respondents were identified through online searches of institutions involved in sexuality education and/or policy-making. Searches were also done to find people of the community who may serve as gatekeepers for sexuality education. Snowballing was also utilized to find and recruit new respondents. Purposive sampling was used to identify the key respondents. Five to six respondents were recruited for each level of the community, as advised by the CRM handbook (Oetting et al., 2014). An invitation to participate in the study was emailed to the respondents, along with a participant information sheet and a Supplementary Factsheet about CSE. Informed written consent was received through email prior to the interview. The interviewer used the fact sheet to describe the CSE and its important aspects to the respondents during the interview. All interviews were recorded and transcribed by a professional transcribing service. Preliminary results of the analysis were shared with the participants during subsequent focus group discussions, not reported for this manuscript. There were no repeat interviews and respondent recruitment was predicated on including key stakeholders. Purposive sampling approach for recruitment aimed at to represent perspectives of multiple stakeholder groups. The number of interviews per stakeholder group was determined using the CRM guidelines rather than a data saturation assessment. (Oetting et al., 2014; Ahmed et al., 2021).

### Rapport building and snowballing

It was anticipated that the study’s topic would be difficult to talk about and some steps were taken to build rapport with respondents (Weller, 2017; Nadeem et al., 2021). When the online call began, the interviewer went over the factsheet and the study’s goal once again. Participants were given time to settle before the recording began. Prior to the recording, they were reminded of the importance of confidentiality/anonymity, and informed that anything communicated will be used solely for research purposes, to further reinforce trust. We were only able to identify and contact 15 possible respondents through internet searches with only 10 responses. Finding potential study participants through internet searches was difficult. Therefore, respondents were asked to recommend participants after the recording had stopped. A total of 28 candidates were recommended and 25 responded.

### Qualitative content analysis

Qualitative content analysis allows to generate inferences from verbal, visual, or written data in an inter-subjective and systematic manner (Bengtsson, 2016). This method was used to analyze and interpret the interview data to explore community views that contribute to resistance around sexuality and to explore implementation strategies. The dataset was coded and maintained using MAXQDA Analytics Pro 2020. To create the initial coding system, an open coding procedure was combined with an inductive manifest analysis approach (Bengtsson, 2016). During the decontextualization stage, FA described and defined each code. JS checked the coding method and segments independently for reliability and trustworthiness after removing the uncoded data segments (Bengtsson, 2016). During the recontextualization stage, the coded segments and system were changed through mutual consensus. The two researchers also produced summaries for each code. The research group then analyzed the summaries of all codes to identify homogeneous group of codes that could be used to triangulate the data for interpretation during the categorization stage (Bengtsson, 2016). After developing the categories, the findings were drafted during the compilation stage (Bengtsson, 2016). To report the findings and develop the manuscript, Consolidated Criteria for Reporting Qualitative Research (COREQ) was used (Tong et al., 2007). Supplementary Table 2 contains the COREQ checklist.

### Network analysis of inter-code relationship

Using code relation data produced by MAXQDA Analytics Pro 2020, the number of times the same interview segment was coded for multiple codes was used to explore the links across codes (Pokorny et al., 2018). This is essentially code overlap for the same interview segments, and we used it to examine how participants spoke about various themes linked to the topic and how interrelated these topics were based on the coding overlap (Pokorny et al., 2018). However, as the code relation statistics fall short of reflecting the complexities of inter-code relationship, network analysis was employed (Pokorny et al., 2018). The inter-code relationship statistics only show the relation of two codes and are based on the frequency of overlap. In comparison, network analysis, for the same statistics, shows intricate interrelationships between all codes that go beyond inter-code relationship matrices. Although both represent relations between pairs of codes, the network graph provides the relation between a given pair of codes in context, and in relation to all the other codes in the network (Pokorny et al., 2018). Each code was treated as a node for the network and undirected edges were used to depict inter-code relationship (Pokorny et al., 2018). Pearson correlation coefficients were computed to determine the strength of the inter-code relationship. Stata 16.0 was used to compute Pearson correlation coefficients and Gephi 0.9.2 for network analysis. The absolute value of Pearson correlation coefficient was used as the weight of the undirected edges to represent the strength of association between nodes/codes (Pokorny et al., 2018). The average weighted degree, which takes into account the weight of all edges connected to a node, is used to determine the size of each node (Jackson, 2010; Powell and Hopkins, 2015; Pokorny et al., 2018). The nodes’ color was determined by modularity class, splitting the network into clusters of highly connected nodes (Jackson, 2010; Powell and Hopkins, 2015; Pokorny et al., 2018). Modularity resolution may be changed to detect a varied number of clusters; raising the resolution decreases the number of clusters and vice versa. We set the modularity resolution to 1.

## Results

### Descriptive statistics

**Figure 1:**
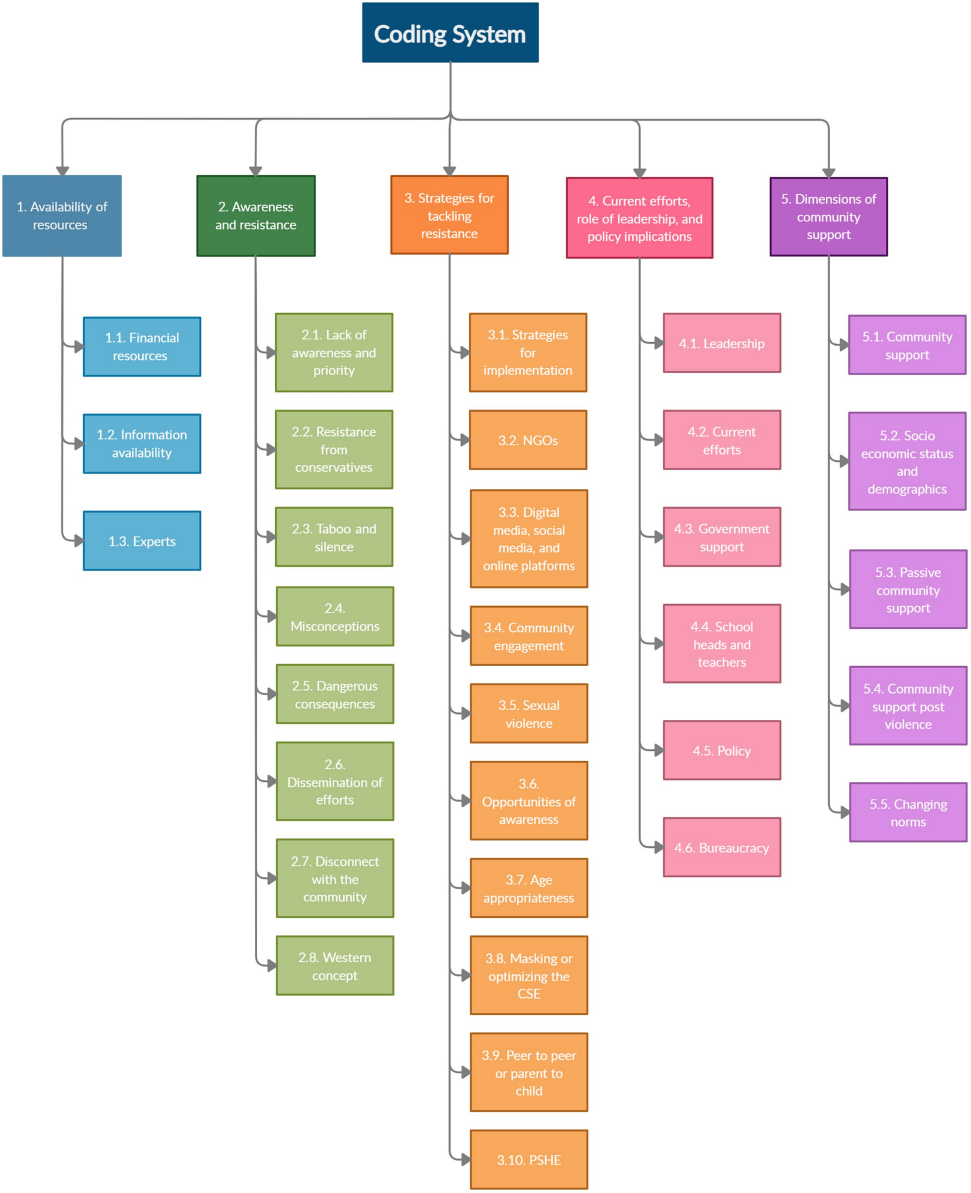
Coding system and categories.

During the categorization phase, five main categories were identified. Supplementary Table 3 contains a portion of the quotes for each code with respondent information and position in the transcript. Figure 1 shows the coding system and categories based on the content analysis. The codes are referred according to the Figure 1 classification in the following sub-sections. Descriptive statistics of the coded segments are provided in Supplementary Table 4. Table 1 shows the respondent shows the respondent category, biological sex, interview duration, and respondent age descriptive statistics.

**Table 1:** Respondent category, biological sex, interview duration, and age descriptive statistics.

Socio ecological tier	Respondent category	Frequency	Percent	Cumulative percentage	
Society	Education Department	1	2.9	11.4	
	Political activist	2	5.7	68.6	
	Health Department	4	11.4	28.6	
Community	Social Media Influencer	2	5.7	77.1	
	News channel	1	2.9	45.7	
	Doctor	2	5.7	8.6	
	Religious Scholar	1	2.9	71.4	
	Community Leader	1	2.9	2.9	
Organizational	NGO	5	14.3	42.9	
	Teacher/Rural	4	11.4	88.6	
	Teacher/Urban	4	11.4	100	
	Head of School	2	5.7	17.1	
Interpersonal	Parent	6	17.1	62.9	
Total respondents		35	100	100	
Biological sex					
Female		20	57.1	57.1	
Male		15	42.9	100	
		Mean	Median	Minimum	Maximum
Interview duration		30.8	29.5	17.5	50.5
Age		31.8	30	23	59

### Availability of resources

There are almost no resources available, so greater support, especially from the government, is necessary. Most community members are unwilling to pay for CSE; however, they may be willing if they understand its importance. Leadership is also financially unsupportive towards CSE. Most funding comes from international donor agencies and goes to NGOs working on such issues (Code 1.1. Financial Resources). The community has access to some information in the form of brochures and media. However, only a fraction of people is reached, and the material on internet is unreliable. Accessible information focuses on sexual abuse and violence rather than CSE (Code 1.2. Information Availability). There are only a few specialists/experts in the community, and even fewer who are skilled in CSE. Some experts act as consultants for NGOs, particularly in adolescent health (Code 1.3. Experts).

Respondent 12, Female, Health Department, Age 36, Pos. 80: “I think that a lot of private NGO’s are being funded on this topic. Yes. There is a lot of international funding available and I think this would continue, but then again I think a lot are not aware of the opportunities available about the resources available. But yes, there is no national level funding on such things, mostly international.”

### Awareness and resistance

Specific understanding is lacking, and numerous misconceptions prevail. Some community members, particularly those who took biology in high school, may be familiar with reproduction concepts. Community must deal with several other challenges, including socio-economic and other health-related concerns. Leadership’s low priority is mainly due to a lack of awareness and prevalence of sexuality-related stereotypes resulting in poor or non-existent allocation of resources (Code 2.1. Lack of awareness and priority).

Respondent 34, Female, Social Media Influencer, Age 38, Pos. 84: “Since they (community) do not know it’s an issue (sexual health), they do not think it’s an issue.”

Religion and cultural traditions have a major role in resistance and opposition. Generally, conservatives are opposed to CSE, and religious leaders are particularly vocal in their resistance (Code 2.2. Resistance from conservatives). They exercise authority and influence over community members, decision-making and priority setting. There are religious and cultural dimensions that contribute to the culture of silence. Although some teachers and parents might discuss it, the broader public may be apprehensive to do so. The perspectives of male and female community members are also at odds. Men, with a contemptuous attitude toward gender issues, dominate decision-making and organizational control. Females consider menstruation as a stigma and often take their menstruating daughters out of school (Code 2.3. Taboo and silence).

Respondent 13, Male, Doctor, Age 35, Pos. 66: “I mean God, people talk America is the most liberal society, where you can voice out opinion. No, you do not know the power of clergy in Pakistan. They can block the road, they can formulate this issue with blasphemy, for their interest, they can manipulate and extrapolate things to a very hazardous level, in a very toxic manner. They can block the roads; they can start violence and agitation… So definitely they can start agitation, here can be lockdown and violent protests.”

Numerous myths and misconceptions circulate regarding CSE. People do not believe that it is beneficial or desirable for their children (Code 2.4. Misconceptions). A common misconception is that CSE will lead to sexual intercourse at an earlier age. If such topics are discussed openly, there may be resistance ranging from verbal abuse to physical assault (Code 2.5. Dangerous consequences).

The initiatives or projects carried out by NGOs and the government face difficulty in dissemination. Normally, organizations publicize their activities but for CSE there may be opposition especially from religious extremists, making it difficult to raise awareness about current activities. Therefore, for some projects, consultation sessions with stakeholders are held at the ministry, to which some religious scholars are invited to get their support (Code 2.6. Dissemination of efforts). The community-based approach is becoming increasingly popular due to better community engagement. However, the community and the implementers/practitioners of health programs are disengaged (Code 2.7. Disconnect with the community). Some community members believe that CSE is a westernized concept and population appears to be resistant to western influences leading to resistance (Code 2.8. Western concept).

Respondent 8, Female, Teacher Rural, Age 23, Pos. 30: “Because they feel (community) a lot of the efforts that have happened, they have been very removed from the people themselves … they are talking in a language that is not very accessible to the students or whoever is listening.”

### Strategies for tackling resistance

Some strategies to deal with resistance to CSE were suggested; the topic should be discussed in a sensitive manner, with evidence, and in line with the cultural/religious sensitivities, and leaders should be involved and given awareness to garner their support (Code 3.1. Strategies for implementation). Some NGOs are working with communities to raise awareness about the issue on the internet by sharing educational content (Code 3.2. NGOs). However, NGOs face difficulty in obtaining NOCs (no objection certificate; Code 3.2. NGOs).

The internet has provided community members access to a lot of information. The digital media platforms, particularly social media, can be used to raise awareness about the issue. Sexual education issues are widely discussed on social media, and social media influencers are playing an important role in raising awareness (Code 3.3. Digital media, social media, and online platforms). Leadership and influencers can play a critical role in engaging community through digital platforms (Code 3.4. Community engagement).

Respondent 1, Female, NGO Employee, Age 27, Pos. 145: “So once that’s shared (content related to CSE), imagine it being shared a million times all over the internet, all over social media. And I would say that forwarding messages, such as in your phone, like WhatsApp and other message hubs, they are a source of connection to parents …… It’s a huge you could say platform, where something like this can be shared.”

There is a lot of sexual violence and a lack of support to prevent or mitigate it. Community members are concerned about their children’s safety and may empathize with the problem. This awareness is also attributed to the initiatives on social media and digital platforms. So, community engagement can be further strengthened by emphasizing children’s safety (Code 3.5. Sexual violence). The media, when used in a positive and strategic manner, can help raise awareness (Code 3.6. Opportunities for awareness).

Respondent 7, Female, NGO employee, Age 30, Pos. 118: “I feel like it (CSE) just needs to be sold to them (community) in a way where they feel like it’s personal and it’s beneficial for them, and it’s not a Western idea or an outsider concept that’s not relevant to them. It has to be adapted in a way and presented to them…. where it feels personal and very relevant to them.”

Many people appear to be unaware of the content’s age-appropriateness leading to many misconceptions, hence, educating about it may be beneficial in overcoming resistance (Code 3.7. Age-appropriateness). It is crucial to sell the topic. Thus, it is important to use acceptable terminology while keeping in mind the local sensitivities, i.e., rebranding for increased acceptability (Code 3.8. Masking or optimizing CSE). Because children trust other students and are hesitant to discuss taboo topics with their parents, peer-to-peer learning can be easier for them. However, it may spread incorrect and potentially harmful information. Parent-to-child learning can also be advantageous for breaking the stigma (Code 3.9. Peer-to-peer or parent-to-child). Some local private schools provide an optional subject, Personal Social & Health Education (PSHE), that covers some aspects of CSE, but very few teachers have the necessary training to deliver the content (Code 3.10. PSHE).

Respondent 18, Female, Teacher Urban, Age 30, Pos. 5: “Keeping this particular fact in mind, private schools have recently started introducing different modules that cover health education, sex education as well. I used to teach pre-O levels in a private school, in Froebel’s and they had recently implemented a subject called PSHE, Personal Social Health Education. I believe it’s also compulsory in the United Kingdom. They had brought actual people who had background in development to help them develop a curriculum for it. There was a very big readiness for it, and they wanted to introduce it to children from grade six onwards, which essentially when you are 11–12 years old. I saw that happening at private schools where a discussion, led by teachers, was initiated for this purpose.”

### Current efforts, role of leadership, and policy implications

The national political leadership appears reluctant to implement CSE. The support level varies depending on the leaders; local community leadership is more supportive than political leadership. Due to religious concerns and the societal stigma, leadership is hesitant to openly endorse and advocate the cause. However, leaders have a critical role in implementing CSE due to their influence. Since leaders have close ties to the community, they must avoid any controversy, particularly one that could be interpreted negatively, such as sexuality. Therefore, they are either indifferent or opposed as their support might not be positively received in the community (Code 4.1. Leadership).

Respondent 18, Female, Teacher Urban, Age 30, Pos. 31: “I think they are (leadership) politically correct in a lot of places, but they do not really like to get their hands dirty.”

A few organizations and individuals are working to raise awareness. Sexual assault and rape are addressed by these organizations, but CSE is usually ignored. Menstrual hygiene, gender issues, contraception, reproductive health, STDs, maternity, and child health are some of the issues NGOs are working on. The National Ministry of Health Coordination and Regulations is also collaborating with NGOs on some programs concentrating on SRH care delivery (Code 4.2. Current efforts).

Government and NGOs collaborate on a range of important issues. As the community engages with these issues, leadership at the Ministry of Health is supportive and certain projects are being implemented, particularly on child abuse, violence, and menstrual hygiene. Due to a lack of community engagement, there is a divide between community leaders and policymakers. The responses were contradictory regarding funding. Some participants stated they do so, but it is not for prevention but to curtail the problem. Furthermore, some stated that the government is making attempts by allocating funds while others argue that no attempt is being made. However, the government’s major focus and priority is on other health and prevention issues (SRH care delivery) leaving CSE with a low priority (Code 4.3. Government support).

Respondent 28, Female, Health Department, Age 26, Pos. 65: “So yes. If you talk about the government, then yes, the government has taken up the initiative of starting up the sexual and reproductive health program across the nation. And most of the interventions of regarding this will be incorporated into the universal health coverage benefit package. That gives an idea of the sustainability of the project, as well as the implementation of the services. So, there is commitment from the political, as well as the healthcare providers.”

Teachers and the community have a close association. Teachers are likely to be supportive of CSE because they have a strong relationship with children and are aware of the negative consequences of a lack of CSE. Spreading awareness in schools is effective, and teachers and school owners may significantly impact CSE implementation. Private schools put in more effort than public schools, but lack of teacher training is a critical obstacle. Teachers also emphasized the importance of developing sexuality content based on the cultural context (Code 4.4. School heads and teachers).

Generally, there is lack of health issues related legislation and policy formulation is needed to increase community’s awareness about the seriousness of the issue. Most policies are dedicated to child abuse and violence, which has gained national attention after the Zainab and Kasur cases of child abuse. However, there are no policies or laws governing CSE, but policymakers may be interested (Code 4.5. Policy). For each municipality, there are area education officers (AEO) who must be onboard prior to any implementation. Due to the risk of stigma, AEO may not get directly involved, but they can help NGOs and get community members involved. If the AEO is a woman, there is a good chance she will give such initiatives a higher priority. District management and the deputy commissioner are also involved in various social issues, although they are not directly interested in CSE (Code 4.6. Bureaucracy).

Respondent 5, Female, Teacher Rural, Age 23, Pos. 52: “For schools, the only party who can make sure that these kinds of seminars are conducted is the area education officer (AEO). For every district there’s a separate AEO…. If an AEO is against situations (initiatives) like these they will make sure that the school principals do not let any seminar be conducted in their schools. The AEO does have that kind of power and authority over the principals. Yeah, that’s the only approval you need…. recently we had an interview with our AEO. She wanted us to get some NGO on board…. she did not reach out to the NGO herself; she put us to the task. That’s usually the way they go…. I do not think they (AEO) want direct involvement with an NGO like that.”

### Dimensions of community support

Support is influenced by several factors. Female leaders are more likely to endorse CSE because they may understand and relate to the cause. Media influencers, entertainers, and leaders lend their support to social media initiatives (Code 5.1. Community support). In terms of sexual education, the perceptions vary based on socio-economic status, age, education, and culture. The issues are increasingly debated on university campuses, in academic circles and in private schools. Sexual education takes a back seat in the poor and middle classes because they must prioritize basic education. In terms of CSE perceptions, there is also a distinction between private and public schools (Code 5.2. Socio-economic status and demographics).

Employee, Age 27, Pos. 4: “It depends on what kind of schools you want the implementation to be in…. So, there are private schools, there are public schools and then there are government schools. In government schools, I would say it would be a tough decision to make, because again lower income people are able to afford this. Middle income families are able to afford government and public schools. But again, government schools have that ideology, you know, that I’ve heard that their syllabus has not even changed over the years since they have started the curriculum. So, kind of changing that perspective about teachers and students there (government schools), even their parents, it will be pretty hard.”

Many people, particularly on Facebook and Twitter, use social media to passively support divisive but important causes like sexuality education, but this is mostly due to its trendy character (Code 5.3. Passive community support). Understanding the difference between bad and good touch, according to the community, is important for children’s safety, protection, and avoiding abuse incidents. When an incidence of child abuse is reported, community support for child-protection programs increases. The Zainab and Kasur cases of child abuse, for example, sparked a national debate on the subject but most forms of support are reactive rather than proactive. However, the internet and digital channels can be a platform for dissemination of information (Code 5.4. Community support post violence). If given the chance, many young people would like to participate in active working groups. However, the elderly and maybe grownups may demonstrate their opposition. (Code 5.5. Changing norms).

Respondent 11, Female, Teacher Urban, Age 42, Pos. 11: “Yeah. Because in the past few years, we had some child abuse cases that the case of Zainab and other ones. After that, I think the awareness is there, and they are people who think that this is important, but at the same time, it is considered to be inappropriate to discuss anything related to adolescent health or sex education in Pakistan. So, they (community) just doing it in a very different way, posting videos. And that would include all the age groups. I came across the videos, and they have several slogans for very young girls, particularly. And in terms of for adults, what I’ve seen is more with the domestic abuse and other kinds of abuse.”

### Network analysis

**Figure 2:**
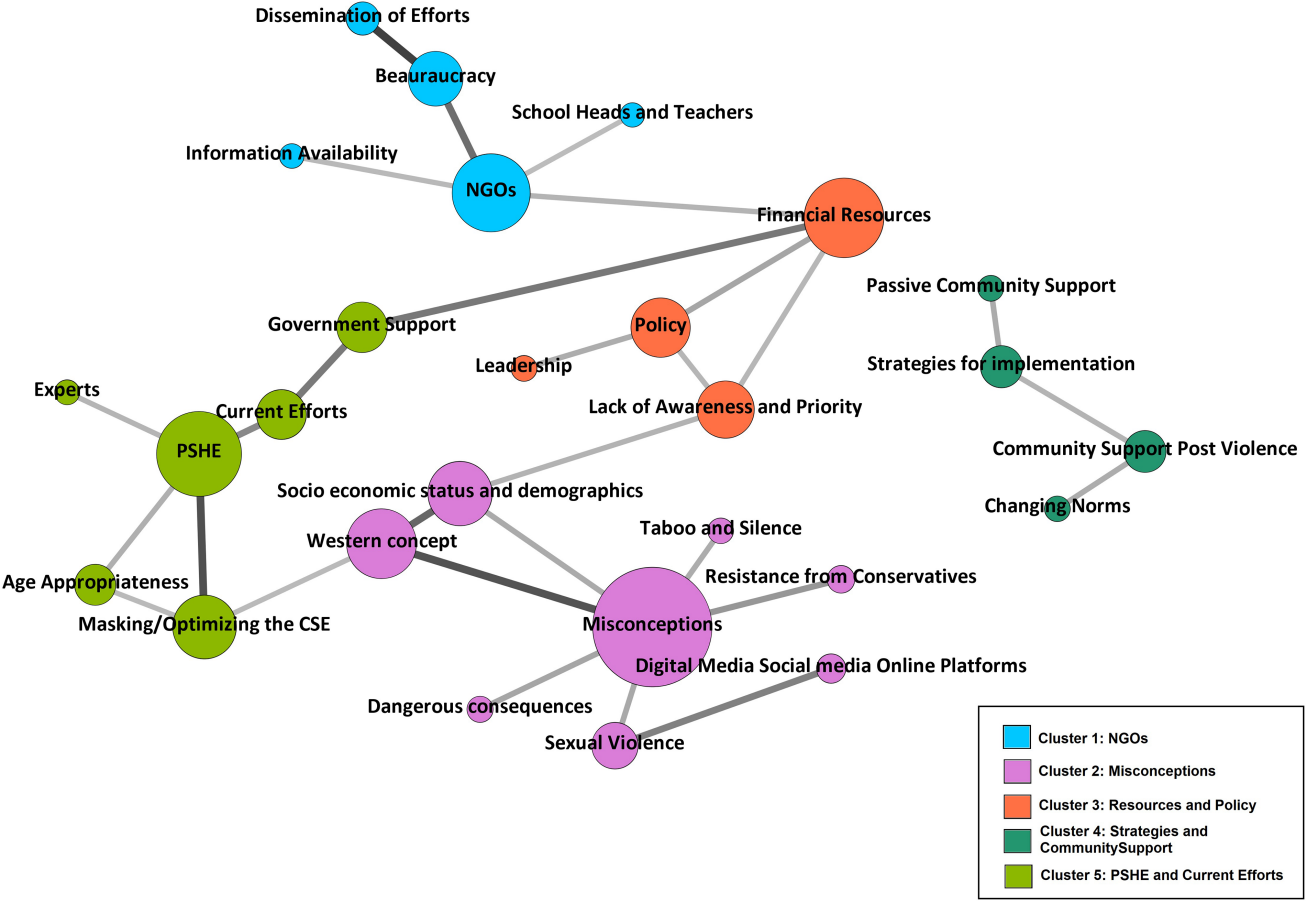
Network analysis and clusters.

Supplementary Tables 5, 6 contain the descriptive statistics for inter-code relationships and the Pearson correlation coefficient matrix, respectively. The network for the inter-code relationship is shown in Figure 2. Due to the large number of undirected edges, detecting relevant linkages was difficult. As a result, Figure 2 only shows statistically significant relationships at the 1% level. The modularity resolution was set to 1, resulting in the identification of five unique clusters of interconnected nodes/codes. The clusters of nodes/codes identified were: NGOs, misconceptions, resources and policy, strategies and community support, and PSHE and current efforts (Figure 2). The size of nodes is based on average weighted degree implying that larger nodes are better connected and correlated within the network. The shade of the edges represents the strength of correlation between the connected pair of nodes/codes, the darker the shade, the stronger the correlation. Figure 1 depicts the nodes that connect distinct clusters, as well as the connections within each cluster, which are of key importance.

Cluster 4 (Strategies and community support) is the only cluster that is not linked to any other cluster, indicating that it does not have statistically significant correlations with any other clusters. Between dissemination of efforts and bureaucracy, PSHE and masking/optimizing the CSE, western concept and socio-economic status and demographics, and misconceptions with western concept were found to be much more correlated with each other as compared to other codes. The network also implies that the most important nodes in the network were financial resources, NGOs, PSHE, and misconceptions, and that implementers should prioritize engaging with or addressing these while focusing on CSE activities. Five codes were excluded from the network analysis as they did not have any linkages at the 1% significance level (Code 2.7. Disconnect with the community, Code 3.4. Community Engagement, Code 3.6. Opportunities for Awareness, Code 3.9. Peer to Peer or Parent to child, Code 5.1. Community Support).

## Discussion

The results of the study indicate that the community is resistant to CSE implementation. Misconceptions, lack of awareness and priority, and absence of dedicated resources are a few of the key implementation challenges. While implementing such programs and attempting to establish a counter-narrative and a case for CSE in the Pakistani context, some strategies can be advantageous.

There are a few initiatives underway, but they are failing to gain traction due to widespread misunderstandings. Only a few members of the community agree that such education is crucial for adolescents and children, and that schools are the ideal setting in which to provide it. Although teachers are reluctant and unsure about teaching CSE, one of the reasons for this is a lack of adequate training. Due to the leadership’s and community’s low priority on the issue, there may not be enough funds. Moreover, without a firm priority and adequate resources, teacher training for adopting CSE in schools is not possible (Ahmed et al., 2021; Keogh et al., 2021).

Increased awareness, community engagement, resolving misconceptions, and rebranding CSE in the local context should be the starting point for establishing prioritization for CSE. However, there is strong opposition from the community and religious clerics which can be addressed by increased engagement and confidence-building initiatives. While several NGOs are working on such challenges, they are unable to disseminate their programs to the public (Chandra-Mouli et al., 2018a, 2018b). For long-term planning and execution of programs aimed at SRH, meaningful stakeholder participation is crucial (Svanemyr et al., 2015). NGOs and government institutions are collaborating on a variety of health programs focusing on reproductive and sexual health. NGOs may provide established infrastructure, network, knowledge, and expertise for future initiatives and health programs that currently have community and stakeholder partnerships in existence. Digital platforms and social media give an opportunity to raise awareness and engage communities on such topics, but there is a lack of research on how to utilize these technologies.

Recently, sexual violence has been discussed extensively on social media. Even the leadership has given sexual violence a higher priority after multiple incidences were highlighted nationally [Alert, 2019; NA passes Zainab Alert Bill after amendments by Senate, n.d., Response and Recovery Act, 2019]. However, the support is reactive, passive, and inconsistent. Nonetheless, the community supports such issues and shows lower resistance. Therefore, such incidents can be strategically utilized for increased awareness and community support. PSHE is taught in some elite private schools but not in public schools (Curriculum | www.pshe-association.org.uk). Teachers also identified a severe deficit in their training to teach CSE and PSHE, which must be addressed to improve school implementation.

Although the political and religious leadership seems to be opposed to CSE, their support is critical due to their influence in the community. Leadership support was also emphasized in a recent UNESCO CSE report suggesting that having leadership on board is critical for determining priorities and sustaining such initiatives (UNESCO, 2021a,b,c). When executing programs, bureaucracy and government agencies are important stakeholder, and no effort can be implemented without their endorsement. NGOs work with the government on certain health issues, yet they face obstacles such as obtaining NOCs and setbacks such as the termination of an agreement in Punjab to implement CSE in 2010 (Svanemyr et al., 2015).

Our study has several strengths and limitations. Since a wide range of stakeholders were involved in the study, an in-depth perspective on CSE implementation was gathered. The qualitative methodology yielded rich data on resistance aspects and potential ways forward. The CRA questionnaire helped capture information on leadership, policy issues, present initiatives, topic awareness, and resources. The qualitative methodology provides in-depth insights, perspectives, and opinions, of respondents but it can be difficult to maintain rigor, researchers’ personal bias, generalizability, and reproducibility. To address this, two coders coded the data to improve rigor and minimize personal bias, while discussion among the research team throughout the categorization stage resulted in triangulating the findings. To ensure rigor, some portion of the interview data and coding system is also provided as Supplementary Table 3. For writing the manuscript, the COREQ guidelines were followed for standardization (Tong et al., 2007). Since the respondents were all from Islamabad, there are geographical limitations to the findings’ generalizability.

With the use of network analysis to visually represent qualitative data, we were able to increase the transparency and rigor of the evidence used to support the research findings, interpretation, and conclusions (Pokorny et al., 2018). Furthermore, because the measures derived from the inter-code relations and network analysis are data-driven, others will be able to duplicate the graphs using the same data (Pokorny et al., 2018). The correlations between dissemination of efforts and bureaucracy, PSHE and masking/optimizing the CSE, western concept and socio-economic status and demographics, and misconceptions with western concept were much stronger. The strong correlations between codes, depicted in the network analysis, and linking it to the qualitative findings, the network analysis complements the qualitative conclusions, as explained further. As revealed by the qualitative findings, the bureaucracy is participating in measures to engage the community through disseminating information to increase support through health ministry programs. PSHE is also taught in some schools and, as an established platform, it could be useful in implementing or integrating CSE through schools. Misconceptions and resistance to CSE, as well as labeling it as a Western agenda, are extremely widespread; qualitative data and network analysis show that these are significantly linked to the socioeconomic status and demographics of the community members. The network analysis also shows that financial resources, NGOs, PSHE, and misconceptions were the most important nodes in the network, and that implementers should prioritize engaging with or addressing these while considering school based implementation.

Even though community support and conversations regarding sexuality have evolved over time in Pakistan, the support is passive and largely online. In terms of support, demographics and socio-economic status also have an influence, which should be considered for designing interventions. To develop content and interventions, the target audience’s norms, perceptions, acceptability, and understanding of sexuality should be thoroughly analyzed. Efforts should be made to rebrand CSE for broader community support and engagement. Since the word sexuality has a negative connotation in the community, rebranding and marketing could be advantageous; prior attempts in Pakistan have resulted in improved support (Svanemyr et al., 2015).

## Recommendations

- Communities’ access to information about CSE is limited, owing to widespread misunderstandings. Future efforts should focus on raising awareness by addressing misconceptions and introducing CSE content while highlighting its spiral and age-appropriate approach.
- Participatory methodologies should be used to include diverse stakeholders, including religious clergy, to generate context-specific material while keeping religious and cultural sensitivities in mind. Indigenously generated material may be better equipped to deal with resistance.
- Due to their extensive expertise and well-established community penetration, previously existing networks, partnerships, and infrastructure, such as NGOs operating actively in communities, should be considered when planning an intervention.
- The internet and online social media platforms such as Facebook, Twitter, Instagram, and YouTube, among others, provide a unique and untapped opportunity to reach and engage community members to raise awareness and readiness for CSE implementation, and influencer marketing may be useful in this regard.
- PSHE is taught in certain private schools, this may provide an opportunity to connect the previously existing curriculum and teachers teaching them to broaden and incorporate CSE contents.
- As indicated by the network analysis bureaucracy, PSHE, masking/optimizing the CSE, western concept, socioeconomic status, and misconceptions are the most important aspects of CSE and must be considered when implementing or planning a community-based intervention.

## Conclusion

Innovative marketing and rebranding are essential for priority setting and community engagement in conservative settings and when confronted with resistance, especially for CSE development and implementation. The results suggest that a lack of awareness and knowledge, and widespread misconceptions about CSE are the primary causes of resistance. Community sensitization through strategic awareness campaigns, involving already established infrastructure and NGOs, endorsement by major stakeholders and decision-makers, and using digital platforms for better dissemination are some of the suggested strategies for implementation.

## Author’s note

As with every research, it is beneficial to understand the research team’s positionality and, as a result, our perspective on the findings. The first and third authors were bilingual Pakistani males proficient in English and Urdu. The first author, who is presently based in Germany as a doctoral researcher and has a background in public health with an emphasis on adolescent SRH, led the data collection and analysis processes. With a background in economics and a PhD from the United States, the third author is based in Pakistan and teaches at a university. The second and fourth authors are both white Germans who are bilingual (English/German). The second author is a woman who works as an early-stage researcher and has a background in public health. A senior researcher male with a background in social epidemiology and gender studies is the fourth researcher. All authors contributed to the interpretation of the study’s findings and implications (details in author contribution section). It is possible; nevertheless, our ethno-racial backgrounds influence how we view the findings. To avoid speaking for the data and remain impartial, especially while analyzing qualitative data, the data was separately assessed by two researchers (more details in methods section). All of the researchers collaborated as a team, holding regular meetings to ensure that the study was directed by their collective cultural experience and skills. This was a collaborative team project that ensured the study was sensitive to and appropriate for the context in which it was carried out with careful diverse stakeholder involvement and perspectives (see section participant details). With the researchers’ diverse ethno-racial backgrounds (Pakistani/German), it was also able to confront the epistemological debate of insider and outsider perspective. Overall, the research team positions itself in favor of CSE implementation in schools due to the evidence base showing CSE’s positive impacts on the well-being of children and adolescents.

## Data availability statement

The raw data supporting the conclusions of this article will be made available by the authors, without undue reservation.

## Ethics statement

The studies involving human participants were reviewed and approved by Pakistan Health Research Council (PHRC) (Reference number: No.4-87/NBC-453/20/1815). The patients/participants provided their written informed consent to participate in this study.

## Author contributions

FA and TB: conceptualization, methodology, resources, and project administration. FA and GA: software and visualization. FA: validation. FA, JS, GA, and TB: formal analysis. FA: investigation and data curation. FA, JS, and TB: writing--original draft preparation. TB: supervision. All authors contributed to the article and approved the submitted version.

## Funding

This work was supported by the Department of Prevention and Evaluation at the Leibniz Institute for Prevention Research and Epidemiology–BIPS and the German Academic Exchange Service (DAAD) as part of its Research grant Doctoral program.

## Conflict of interest

The authors declare that the research was conducted in the absence of any commercial or financial relationships that could be construed as a potential conflict of interest.

## Publisher’s note

All claims expressed in this article are solely those of the authors and do not necessarily represent those of their affiliated organizations, or those of the publisher, the editors and the reviewers. Any product that may be evaluated in this article, or claim that may be made by its manufacturer, is not guaranteed or endorsed by the publisher.
