# Supplementary material for: Understanding community resistance to sexuality education and exploring prospective implementation strategies in Pakistan: A content and network analysis of qualitative data
Source: Front Psychol. 2022 Sep 15;13:864465. doi: 10.3389/fpsyg.2022.864465 (PMC9521423; doi:10.3389/fpsyg.2022.864465)
Supplement: Supplementary file 1 [file Data_Sheet_1.PDF]

# Supplementary Questionnaire 1: Community Readiness Assessment for implementing school-based health and reproductive health syllabus for youth in Islamabad.

*Required

1. **Sex ***
   *Mark only one oval.*

   - Male
   - Female

2. **Work title/occupation ***

3. **Age ***

4. **Do you live in the community? ***
   *Mark only one oval.*

   - Yes No

5. **How long have you lived in the community? Years ***

6. **Do you work in the community? ***
   *Mark only one oval.*

   - Yes No

7. **Email ***

8. **Contact Number ***

# Community Readiness Interview Questions

For the following questions, please answers keeping in mind your perspective of what community members believe and not what you personally believe.

**1. On a scale from 1-10, how much of a concern is (youth reproductive health education and other socio-health related topics in school/college syllabus) to members of (Islamabad/Community), with 1 being "not a concern at all" and 10 being "a very great concern"?**

*Mark only one oval.*

| | 1 | 2 | 3 | 4 | 5 | 6 | 7 | 8 | 9 | 10 | |
|---|---|---|---|---|---|---|---|---|---|---|---|
| **Not of concern at all** | ◯ | ◯ | ◯ | ◯ | ◯ | ◯ | ◯ | ◯ | ◯ | ◯ | **A very great concern** |

**Can you tell me why you think it's at that level?**

# COMMUNITY KNOWLEDGE OF EFFORTS

I'm going to ask you about current community efforts to address (youth reproductive health education and other related topics in school/college syllabus). By efforts, I mean any programs, activities, or services in your community that address (youth reproductive health education and other related topics in school/college syllabus).

**2. Are there efforts in (Islamabad/Community) that address (youth reproductive health education and other related topics in school/college syllabus)? If yes, continue to question 3; if No, skip to question 16.**

*Mark only one oval.*

◯ Yes No

◯

**3. Can you briefly describe each of these?**

**4. How long has each of these efforts been going on? Probe for each program/activity.**

**5. Who does each of these efforts serve (e.g., a certain age group, ethnicity, etc.)?**

**6. About how many community members are aware of each of the following aspects of the efforts - none, a few, some, many, or most?**

*Mark only one oval per row.*

| | None | A few | Some | Many | Most |
|---|---|---|---|---|---|
| Have heard of efforts? | | | | | |
| Can name efforts? | | | | | |
| Know the purpose of efforts? | | | | | |
| Know who the efforts are for? | | | | | |
| Know how the efforts work? (E.g. activities or how they are implemented) | | | | | |
| Know the effectiveness of the efforts? | | | | | |

**7. Thinking back to your answers, why do you think members of your community have this amount of knowledge?**

**8 (a). Are there misconceptions or incorrect information among community members about the current efforts?**

Yes No

**8 (b). If yes: What are these?**

**9. How do community members learn about the current efforts?**

**10. Do community members view current efforts as successful? (Probe: What do community members like about these programs? What don't they like?)**

**11. What are the obstacles to individuals participating in these efforts?**

**12. What are the strengths of these efforts?**

**13. What are the weaknesses of these efforts?**

**14. Are the evaluation results being used to make changes in efforts or to start new ones?**

**15. What planning for additional efforts to address (youth reproductive health education and other related topics in school/college syllabus) is going on in (Islamabad/Community)?**

**16. Is anyone in (Islamabad/Community) trying to get something started to address (youth reproductive health education and other related topics in school/college syllabus)? Can you tell me about that? (Only ask #16 if the respondent answered "No" to #2 or was unsure)**

# LEADERSHIP

I'm going to ask you how the leadership in (Islamabad/Community) perceives (youth reproductive health education and other related topics in school/college syllabus). By leadership, we are referring to those who could affect the outcome of this issue and those who have influence in the community and/or who lead the community in helping it achieve its goals.

**17 a. Using a scale from 1-10, how much of a concern is (youth reproductive health education and other related topics in school/college syllabus) to the leadership of (Islamabad/Community), with 1 being "not a concern at all" and 10 being "a very great concern"?**

*Mark only one oval.*

| | 1 | 2 | 3 | 4 | 5 | 6 | 7 | 8 | 9 | 10 | |
|---|---|---|---|---|---|---|---|---|---|---|---|
| Not a concern at all | ◯ | ◯ | ◯ | ◯ | ◯ | ◯ | ◯ | ◯ | ◯ | ◯ | A very great concern |

**17 b. How much of a priority is addressing this (youth reproductive health education and other related topics in school/college syllabus) to leadership? Can you explain why you say this?**

**18 a. I'm going to read a list of ways that leadership might show its support or lack of support for efforts to address (youth reproductive health education and other related topics in school/college syllabus). At least passively support efforts without necessarily being active in that support?**

**18 b. Participate in developing, improving or implementing efforts, for example by being a member of a group that is working toward these efforts?**

**18 c. Support allocating resources to fund community efforts?**

**18 d. Play a key role as a leader or driving force in planning, developing or implementing efforts? (Prompt: How do they dothat?)**

**18 e. Play a key role in ensuring the long-term viability of community efforts, for example by allocating long-term funding?**

**19 a. Does the leadership support expanded efforts in the community to address (youth reproductive health education and other related topics in school/college syllabus)?**

*Mark only one oval.*

- Yes
- No

**19 b. If yes: How do they show this support? For example, by passively supporting, by being involved in developing the efforts, or by being a driving force or key player in achieving these expanded efforts?**

**20. Who are leaders that are supportive of addressing this issue in your community?**

**21. Are there leaders who might oppose addressing (youth reproductive health education and other related topics in school/college syllabus)? How do they show their opposition?**

## Community Climate

For the following questions, again please answer keeping in mind your perspective of what community members believe and not what you personally believe.

**22. How much of a priority is addressing this issue to community members? Can you explain your answer?**

**23. I'm going to read a list of ways that community members might show their support or their lack of support for community efforts to address (issue).**

**23 (a). Can you please tell me whether none, a few, some, many or most community members would or do show their support in this way? Also, feel free to explain your responses as we move through the list.How many community members…**

*Mark only one oval per row.*

| | None | A few | Some | Many | Most |
|---|---|---|---|---|---|
| At least passively support community efforts without being active in that support? | | | | | |
| Play a key role as a leader or driving force in planning, developing or implementing efforts? (prompt: How do they do that?) | | | | | |
| Participate in developing, improving or implementing efforts, for example by attending group meetings that are working toward these efforts? | | | | | |
| Are willing to pay more (for example, in taxes) to help fund community efforts? | | | | | |

**24. About how many community members would support expanding efforts in the community to address (issue)? Would you say none, a few, some, many or most?**

*Mark only one oval per row.*

| | None | A few | Some | Many | Most |
|---|---|---|---|---|---|
| Community members would support expanding efforts in the community to address (issue) | | | | | |

**24 (a). How might they show this support? For example, by passively than none: supporting or by being actively involved in developing the efforts?**

**26. Are there ever any circumstances in which members of (community) might think that this issue should be tolerated? Please explain.**

**27. Describe (community).**

## KNOWLEDGE ABOUT THE ISSUE

**28. On a scale of 1 to 10 where a 1 is no knowledge and a 10 is detailed knowledge, how much do community members know about (youth reproductive health education and other related topics in school/college syllabus)?**

*Mark only one oval.*

| | 1 | 2 | 3 | 4 | 5 | 6 | 7 | 8 | 9 | 10 | |
|---|---|---|---|---|---|---|---|---|---|---|---|
| No knowledge | ⬭ | ⬭ | ⬭ | ⬭ | ⬭ | ⬭ | ⬭ | ⬭ | ⬭ | ⬭ | Detailed Knowledge |

**29. Would you say that community members know nothing, a little, some or a lot about each of the following as they pertain to (youth reproductive health education and other related topics in school/college syllabus)? (After each item, have them answer.)**

*Mark only one oval per row.*

| | Know nothing | A little | Some | A lot |
|---|---|---|---|---|
| Topics and contents | | | | |
| Benefits of school based Adolescent health interventions | ⬭ | ⬭ | ⬭ | ⬭ |
| General issues regarding adolescent health in the community | ⬭ | ⬭ | ⬭ | ⬭ |
| How adolescent health can be improved | ⬭ | ⬭ | ⬭ | ⬭ |
| Information regarding age appropriateness of adolescent health syllabus | ⬭ | ⬭ | ⬭ | ⬭ |

**30. What are the misconceptions among community members about (youth reproductive health education and other related topics in school/college syllabus), e.g., why it occurs, how much it occurs locally, or what the consequences are?**

**31. What type of information is available in (Islamabad/Community) about (youth reproductive health education and other related topics in school/college syllabus) (e.g. newspaper articles, brochures, posters)? If they list information, ask: Do community members access and/or use this information?**

## RESOURCES FOR EFFORTS (time, money, people, space, etc.)

**32. How are current efforts funded? Is this funding likely to continue into the future?**

**33. I'm now going to read you a list of resources that could be used to address (youth reproductive health education and other related topics in school/college syllabus) in your community. For each of these, please indicate whether there is none, a little, some or a lot of that resource available in your community that could be used to address (youth reproductive health education and other related topics in school/college syllabus)?**

*Mark only one oval per row.*

| | None | A little | Some | A lot |
|---|---|---|---|---|
| Volunteers | | | | |
| Financial donations from organizations and/or businesses | ◯ | ◯ | ◯ | ◯ |
| Grant Funding | ◯ | ◯ | ◯ | ◯ |
| Experts | ◯ | ◯ | ◯ | ◯ |
| Space | ◯ | ◯ | ◯ | ◯ |

**34. Would community members and leadership support using these resources to address (youth reproductive health education and other related topics in school/college syllabus)? Please explain.**

**35. On a scale of 1 to 5, where 1 is no effort and 5 is a great effort, how much effort are community members and/or leadership putting into doing each of the following things to increase the resources going toward addressing (youth reproductive health education and other related topics in school/college syllabus) in yourcommunity?**

*Mark only one oval per row.*

| | 1 (No effort) | 2 | 3 | 4 | 5 (Great Effort) |
|---|---|---|---|---|---|
| Seeking volunteers for current or future efforts to address (youth reproductive health education and other related topics in school/college syllabus) in the community. | ◯ | | | | ◯ |
| Soliciting donations from businesses or other organizations to fund current or expanded community efforts. | ◯ | | | | ◯ |
| Writing grant proposals to obtain funding to address (youth reproductive health education and other related topics in school/college syllabus) in the community. | ◯ | | | | ◯ |
| Training community members to become experts. | ◯ | | | | ◯ |
| Recruiting experts to the community. | ◯ | | | | ◯ |

**36. Are you aware of any proposals or action plans that have been submitted for funding to address (youth reproductive health education and other related topics in school/college syllabus) in (Islamabad/Community)? If yes, please explain.**

# Additional policy-related questions

**37. What formal or informal policies, practices and laws related to this issue are in place in your community? (Prompt: An example of —formal would be established policies of schools, police, or courts. An example of —informal would be similar to the police not responding to calls from a particular part of town.)**

**38. Are there segments of the community for which these policies, practices and laws may not apply, for example, due to socioeconomic status, ethnicity, age?**

**39. Is there a need to expand these policies, practices and laws? If so, are there plans to expand them? Please explain.**

**40. How does the community view these policies, practices and laws?**

**What are the possible strategies to tackle community resistance and increase readiness at the following levels?**

45. **COMMUNITY KNOWLEDGE OF EFFORTS**

46. **LEADERSHIP**

47. **KNOWLEDGE ABOUT THE ISSUE**

48. **RESOURCES FOR EFFORTS (time, money, people, space, etc.)**

49. **Policy makers**

50. **Parents**

58. **Religious Leaders**

Supplementary Table 2: COREQ Checklist

| Topic | Item No. | Guide Questions/Description | Reported on |
|---|---|---|---|
| **Domain 1: Research team and reflexivity** | | | |
| *Personal characteristics* | | | |
| Interviewer/facilitator | 1 | Which author/s conducted the interview or focus group? | Section 2.1. |
| Credentials | 2 | What were the researcher's credentials? E.g., PhD, MD | Section 2.1. |
| Occupation | 3 | What was their occupation at the time of the study? | Section 2.1. |
| Gender | 4 | Was the researcher male or female? | Section 2.1. |
| Experience and training | 5 | What experience or training did the researcher have? | Section 2.1. |
| *Relationship with participants* | | | |
| Relationship established | 6 | Was a relationship established prior to study commencement? | Section 2.3. |
| Participant knowledge of the interviewer | 7 | What did the participants know about the researcher? e.g., personal goals, reasons for doing the research | Section 2.2. |
| Interviewer characteristics | 8 | What characteristics were reported about the inter viewer/facilitator? e.g., Bias, assumptions, reasons, and interests in the research topic | Section 2.3. |
| **Domain 2: Study design** | | | |
| *Theoretical framework* | | | |
| Methodological orientation and Theory | 9 | What methodological orientation was stated to underpin the study? e.g., grounded theory, discourse analysis, ethnography, phenomenology, content analysis | Section 2.4. |
| *Participant selection* | | | |
| Sampling | 10 | How were participants selected? e.g., purposive, convenience, consecutive, snowball | Section 2.2. |
| Method of approach | 11 | How were participants approached? e.g., face-to-face, telephone, mail, email | Section 2.2. |
| Sample size | 12 | How many participants were in the study? | Section 3.1. |
| Non-participation | 13 | How many people refused to participate or dropped out? Reasons? | Section 2.3. |
| *Setting* | | | |

| | | | |
|---|---|---|---|
| Setting of data collection | 14 | Where was the data collected? e.g., home, clinic, workplace | Section 2.2. |
| Presence of non-participants | 15 | Was anyone else present besides the participants and researchers? | Section 2.2. |
| Description of sample | 16 | What are the important characteristics of the sample? e.g., demographic<br>data, date | Section 3.1. |
| *Data collection* | | | |
| Interview guide | 17 | Were questions, prompts, guides provided by the authors? Was it pilot<br>tested? | Section 2.2. |
| Repeat interviews | 18 | Were repeat inter views carried out? If yes, how many? | Section 2.2. |
| Audio/visual recording | 19 | Did the research use audio or visual recording to collect the data? | Section 2.2. |
| Field notes | 20 | Were field notes made during and/or after the interview or focus group? | Section 2.2. |
| Duration | 21 | What was the duration of the inter views or focus group? | Section 3.1. |
| Data saturation | 22 | Was data saturation discussed? | Section 2.2. |
| Transcripts returned | 23 | Were transcripts returned to participants for comment and/or | Section 2.2. |
| | | correction? | |
| **Domain 3: analysis and findings** | | | |
| *Data analysis* | | | |
| Number of data coders | 24 | How many data coders coded the data? | Section 2.4. |
| Description of the coding tree | 25 | Did authors provide a description of the coding tree? | Section 3.1. |
| Derivation of themes | 26 | Were themes identified in advance or derived from the data? | Section 2.4. |
| Software | 27 | What software, if applicable, was used to manage the data? | Section 2.4. |
| Participant checking | 28 | Did participants provide feedback on the findings? | Section 2.2. |
| *Reporting* | | | |
| Quotations presented | 29 | Were participant quotations presented to illustrate the themes/findings?<br>Was each quotation identified? e.g., participant number | Section 3.1. |
| Data and findings consistent | 30 | Was there consistency between the data presented and the findings? | Section 3.1. |
| Clarity of major themes | 31 | Were major themes clearly presented in the findings? | Section 3. |
| Clarity of minor themes | 32 | Is there a description of diverse cases or discussion of minor themes? | Section 3. |

## Supplementary Table 3: Quotations, categories, codes, and respondent information

| Category | Code | Respondent | Coded Segments |
|---|---|---|---|
| 1. Availability of resources | 1.1. Financial resources | Respondent 11, Female, Teacher Urban, Age 42, Pos. 107-108 | Interviewer: Writing grant proposals to obtain funding? Interviewee: Maybe certain NGOs are doing it, but it's not shared. For one thing with public, those kinds of things are never shared within public, but I believe it's happening but a small group of people or NGOs might be doing it. |
| | | Respondent 12, Female, Health Department, Age 36, Pos. 80 | Interviewee: I think that a lot of private NGO's are being funded on this topic. Yes. There is a lot of international funding available and I think this would continue, but then again I think a lot are not aware of the opportunities available about the resources available. But yes, there is no national level funding on such things, mostly international. |
| | | Respondent 13, Male, Doctor, Age 35, Pos. 102 | Interviewee: It depends upon the volunteers, how they put up their stance. Are they confident enough in highlighting this issue and bringing up into the designed attention by the elite society, who are willing to spend. If I had been a volunteer, I mean, I'm not trying to blow my own trumpet, but yes, if I had been an active member and considering the business community, I would have raised some good funds. |
| | | Respondent 15, Female, Health Department, Age 31, Pos. 92 | Interviewee: I'm not sure, but as far as I think, I don't think I would… I don't think sex education is getting any funding. |
| | | Respondent 19, Male, Parent, Age 32, Pos. 33-34 | Interviewer: How many community members are willing to pay more, for example, in terms of taxes or donations to help fund community efforts? Interviewee: If they know that the effort is going in the right direction, if it's not being wasted, or if the money is not being abused, they will definitely pay for this. At least in Islamabad. |
| | | Respondent 2, Male, Teacher Urban, Age 29, Pos. 106 | Interviewee: I'm not aware of any funding by the government. I think any efforts that might be taking place will be coming through NGO's and private institutions. And quite likely I would expect those to continue into the future. |
| | | Respondent 24, Female, Health Department, Age 32, Pos. 33-34 | Interviewer: And do they support in allocating resources or funding to fund such efforts? Interviewee: Yes, for example, regarding SRHR project, we are getting resources from USAID and from WHO also, so a lot of them are agreeing and they are showing highly interest to support this project. |
| | | Respondent 28, Female, Health Department, Age 26, Pos. 67 | Interviewee: I guess very few, very few would be there. Because my reason for saying that is the volunteers must themselves first be, you know, have a very unbiased approach and should be educated enough to convey the message in a proper sense. Because as I said earlier, this would be, this is an issue in itself a barrier that if the education and the knowledge regarding this is not conveyed in its true essence, or is interpreted in a wrong way by the audience or the or by the population, the education intervention is subjective. So very little, yeah. |
| | 1.2. Information Availability | Respondent 11, Female, Teacher Urban, Age 42, Pos. 86 | Yeah, it's available. There are few brochures. There was one time that I came across a brochure as well in terms of they showed a figure being black and it showed that no people should be coming across particularly with respect to child sexual abuse. |
| | | Respondent 16, Female, Parent, Age 31, Pos. 61 | Interviewee: Online people have access to everything online, we do like, I, myself, if I have an issue, I don't know how to address my kids. So if I have a problem, I go, I Google it. I find a video and I address the problem accordingly. So we have.......because time again and again there are cases of child abuse, child violence……..We have news articles on it. We have posts on it……but people, they can go and they can read them on their own way. |
| | | Respondent 21, Female, Parent, Age 35, Pos. 56 | Interviewee: See, I haven't come across any brochure or pamphlet. But there might be some articles available online, not accessible to many, I would say only to those who come across them and read them on a regular basis. So, I've touched the brochure, and so, yeah, that's it, nothing is mentioned otherwise on any television level. Nothing like that. |
| | | Respondent 28, Female, Health Department, Age 26, Pos. 62-63 | Interviewee: No people don't usually actively look up for it, because I might have a bias opinion about it, because I haven't heard any of like my colleagues or my friends or my social circle, or even myself, I've never looked up for stuff like this, even… sorry, despite the fact that I'm working in the health sector. So yeah, I would rather say no. |
| | | Respondent 29, Female, Head of School, Age 29, Pos. 64-65 | Interviewee: Honestly, I'm not sure. I mean, I know that Greenstar does something, but I'm really, really not sure what do they do. So I mean, if it's there than it's not very… it's not like very common or just out there for everyone to read or it's not common in the society, so I'm not sure. |
| | 1.3. Experts | Respondent 28, Female, Health Department, Age 26, Pos. 74-75 | Interviewer: Experts, who can deliver sexuality education to the community?<br><br>Interviewee: So I guess there would… there would be a need for training the master trainers. So I don't guess right now, but this is an area to be worked upon. |
| | | Respondent 31, Male, Political Activist, Age 31, Pos. 79-80 | Interviewee: You know, there would be certain expert, but still, you know, this is an ongoing process, learning is an ongoing process. So right now, even there are experts, but they would be on a like very, you know, very limited scale. So this has to be taken as a, you know, a full launched campaign across the country. And where every tier of the, you know, community should be participating in their own capacities. |
| | | Respondent 32, Male, Doctor, Age 40, Pos. 76-77 | Interviewee: They're very few experts regarding adolescent and child health in the country. I mean, so there are a few people not much, a handful of 10 to 12 professionals…........have done masters in the relevant field and research work…… there are not many professionals available. |
| | | Respondent 34, Female, Social Media Influencer, Age 38, Pos. 101-104 | Interviewee: I don't think any institutes or universities in which you can actually go and study sexual health in Pakistan. So there might be one or two universities that were … teacher like public health, sexual health or gender health, but not a lot. So, yeah, |
| | | Respondent 6, Female, Community Leader, Age 38, Pos. 128-129 | Interviewee: They have. They have experts, yes. Experts in the sense that they even have PhDs in adolescent health and all. Rozan definitely does, but I think Sahil was taking consultation from somebody. I'm not sure who. I didn't get the details but, yeah, I've heard. |
| | 2.1. Lack of awareness and priority | Respondent 1, Female, NGO Employee, Age 27, Pos. 15 | I don't know about any such groups. I only know that you brought up the topic first and that's how I know this is an important subject that should be instilled in schools in colleges, but up till now, I haven't heard of any organization or people that are working towards this cause. |
| | | Respondent 27, Male, Religious Scholar, Age 31, Pos. 28 | Interviewee: We don't give it much of a thought actually. Maybe because we have a lot of things in our mind, like, finances is a big issue for us. A child who grows up he's always wondering what he will do in his life, what will be his earning? His parents are always after him. Maybe the parents think that their children will support them when they grow old. I think we don't give it much importance. |
| | | Respondent 28, Female, Health Department, Age 26, Pos. 46-47 | Interviewee: Not… I would rather say no, not at all, zero. Because personally I belong to the health sector and I did not have a clue about it. So a very, very less number of people, who are specifically working towards this agenda might know about it, but overall and rate it zero. |
| | | Respondent 29, Female, Head of School, Age 29, Pos. 21 | Interviewee: Yeah. I mean I know of some people who might be willing, you know, like advocates of this issue, but there are just so many other issues right now in the society that whenever they do sort of come to the idea of, you know, like when they to actually think about doing something about it, there are just so many other issues at hand first that they just believe that even when just providing basic education to this age group is the first step. |
| | | Respondent 29, Female, Head of School, Age 29, Pos. 45 | Interviewee: Unless you're a biology student or something related to medical health, or just a health professional, anything if you've had, if you have not studied biology in your school, you do not know what's actually happening, for sure. I mean, I have friends, you know, have studied different subjects and they have… they don't know how many… like they don't know what's happening in the body whatsoever. So they don't know why women have periods, they don't know how STD work. And they don't know what sexual diseases are there. It's just, it's a mess, all of it as a mess. So there's no awareness of anything. |
| | | Respondent 34, Female, Social Media Influencer, Age 38, Pos. 84 | Interviewee: Since the don't know it's an issue, they don't think it's an issue. |
| | | Respondent 35, Male, NGO Employee, Age 51, Pos. 46 | And because of a lack of knowledge, they don't like to discuss these things among the people, within the community. But one thing is sure, everybody wants … everybody wants their people, their kids should know about what is wrong, what is right. |
| | | Respondent 4, Female, Teacher Urban, Age 24, Pos. 80 | Interviewee: I don't think it's very high on the priority list, especially given circumstances. Like, because of the, COVID-19 there so much pressure on like fixing the economy and helping local businesses. |
| | | Respondent 9, Male, Teacher Rural, Age 23, Pos. 2 | For my school's community, I think that curriculum-based education it's not a priority right now. It's around 3 because some parents are concerned about such things, gender violence, sexual health, and a lot of things, but it's not a priority for them right now. |
| | 2.2. Resistance from conservatives | Respondent 1, Female, NGO Employee, Age 27, Pos. 41-45 | Interviewee: So liberalism in Pakistan has increased over the years, but side-by-side conservatism has also been increasing exponentially……. if they get to know that this kind of topic is being spread among the youth of Pakistan, it would be a completely negative turn point for them, because they would say this is against our religion and against the religion of Islam basically. So I think that they would not be agreeing to this sort of implementation in schools and colleges. |
| | | Respondent 10, Male, Political Activist, Age 30, Pos. 7 | Also, is that for most of these communities, obviously, they are molded by what the larger norms of society are. And this community, already underprivileged community, they can't break free of those norms as some more privileged people are able to. So, they're very entrapped by what the larger society are, which is, obviously, very conservative. To put it in a way. |
| | | Respondent 10, Male, Political Activist, Age 30, Pos. 40 | There are some times, especially for women, oftentimes we talk to women, especially when they're going alone, to participate in efforts, sometimes because it is often very patriarchal societies, and sometimes from religious leaders as well. It's been women, especially when they're going alone to participate in efforts, sometimes because it is often a very patriarchal societies and sometimes from religious leaders as well. |
| | | Respondent 10, Male, Political Activist, Age 30, Pos. 81 | There's very few but again, some of them (conservatives) hold a lot of -- not political but social power, they have a lot of social capital. Usually sometimes, their social capital is enough for the -- climate at that time is hostile enough that they're able to succeed. There's few of them, but they will as a substantial amount of social capital. You would see sometimes that these interventions would fail when one of these would speak up. |
| | | Respondent 18, Female, Teacher Urban, Age 30, Pos. 20 | Interviewee: I would say family pressure, maybe. Religious pressure also. The city has certain pockets— I wouldn't say all of it— but it does have certain pockets that are extremely hard line, religious hardliner, very averse to all forms of modern education and science. |
| | | Respondent 18, Female, Teacher Urban, Age 30, Pos. 41 | Interviewee: As it is with all things Pakistan, there is a community… I would put it at the uneducated hardliner religious groups; most of them extremely right-wing organizations. Within Islamabad there's not a large amount of them. They have more of a presence in different areas of the country……In Islamabad you can actually count on your fingertips two or three very, very hardliner religious organizations. They have their sway and they have their say. |
| | | Respondent 2, Male, Teacher Urban, Age 29, Pos. 18 | They might. Yeah, they might. If, I mean, again, it's all about how the… what kind of religious backlash there is to such a thing. I mean, we are talking about a country, where polio vaccination workers, they get attacked. So it all depends on the kind of religious backlash that they expect or the religious backlash that they receive. |
| | | Respondent 2, Male, Teacher Urban, Age 29, Pos. 38 | So the way they will demonstrate their feelings towards these sex education or any kind of education regarding sex, they are going to block the main highway. They are going to stay in here for weeks and they are just going to disrupt the lives of millions of people. |
| | | Respondent 20, Male, News Reporter, Age 30, Pos. 58 | Interviewee: Well, yes. There are many people, I guess, people who oppose the ideas in the community. They come from different segments. One of them is probably the clergy. They use their very circle of influence to oppose the matter. The other one could be the MPs belonging to, or local leadership belonging to parties which have conservative political ideologies. Because they have to play to the electorate, so they would be opposing the ideas as well. |
| | | Respondent 25, Male, NGO Employee, Age 29, Pos. 48 | Interviewee: In an ideal world, there wouldn't be but in Pakistan, as of 2020, I feel that the fundamentalist right or the conservative right, I would more specifically say the conservative right, because they don't know the entire picture because they're more driven by what their community tells them rather than doing research themselves, I feel that that influences their decision and that's why they're rigidly opposing it. I feel that it is ignorance on their part, which prevents them from integrating this aspect into their community and for the betterment of their people. |
| | 2.3. Taboo and | Respondent 10, Male, Political Activist, Age 30, Pos. 75 | That varies. So, you'd often see a lot of women, for example, attending, not a lot of men. Again, there's a sharp divide. So, it's mostly, adult women who will be attending these. But I'd say a significant portion of them. So, significant like in any given community, for example, you easily see 50% of the women attending some intervention like this or some something like this happens. |
| | | Respondent 11, Female, Teacher Urban, Age 42, Pos. 23-24 | Interviewee: Another thing, again, it's a social stigma here. And as I said even parents are not going to educate their own kids which is like, against, warn them that anything related to sex, they're not going to educate them, they're not going to tell them because even within the same families, it's considered to be something which is not talked about. |
| | | Respondent 15, Female, Health Department, Age 31, Pos. 10 | Interviewee: Probably would because of the stigma associated with sex education and the taboo that's associated with it in our society. |
| | | Respondent 15, Female, Health Department, Age 31, Pos. 107 | Interviewee: I think they'd receive a lot of opposition from a lot of community leaders, from religious leaders and also parents themselves. Cause it's considered taboo……….it's hushed in the society. |
| | | Respondent 23, Female, Parent, Age 29, Pos. 4 | Interviewer: It's a stigma around the community. |
| | | Respondent 29, Female, Head of School, Age 29, Pos. 57 | Interviewee: Because the thing is, it's easier to live in denial than to actually acknowledge it. So even though there is a lot of literature out there to see what could be done for it, it's just easier to live in bliss of it. So yeah, they wouldn't do it. |

| | | | |
|---|---|---|---|
| 2. Awareness and resistance | 2.3. Taboo and silence | Respondent 32, Male, Doctor, Age 40, Pos. 9 | The girls, they have to be told in time about the period coming. And they have to be told about their body, how to take care of their bodies……. I mean, I grew up in a very educated household, so I was told… I was told about sexuality and I was uncomfortable hearing that from a parent, but I was told, so I can't say it, yeah, I wasn't able to. But other than that, they were many of the people in my class who weren't told, it was a total shock to them. |
| | | Respondent 34, Female, Social Media Influencer, Age 38, Pos. 11-12 | Interviewee: Menstruation, yeah (we were taught about it). And all my other friends and people who I grew up with, even my cousins, haven't heard about it………. So I feel no, there's nothing like that happening in Islamabad, no. |
| | | Respondent 35, Male, NGO Employee, Age 51, Pos. 14 | People don't like to talk about sexual education, especially they don't like to send their kids for this type of educational activity. |
| | | Respondent 35, Male, NGO Employee, Age 51, Pos. 30 | But again, the problem is there, people don't like to teach their kids about sex, people don't take any courage, people don't have any courage to teach their skills about sex. It's a difficult thing. |
| | | Respondent 5, Female, Teacher Rural, Age 23, Pos. 36 | Interviewee: Once you actually get to talk to them, this is… We haven't had Rozan come to our school yet, but some other fellows who have had Rozan come to their schools, the mothers are really apprehensive in the start. Then when they see that they're actually giving their girls hygiene kits and they're stocking up the bathrooms with the essentials, it kind of, I think, makes it better because they know this is something that's going to happen. I think eventually they get used to the idea that okay, their girls are being helped out and they're a lot more comfortable coming to school. Eventually I think they warm up to it and then they appreciate it as well in the long-term, but initially they're very apprehensive about the whole thing. |
| | 2.4. Misconceptions | Respondent 1, Female, NGO Employee, Age 27, Pos. 110 | And according to our religion, it's not the right thing to do, you know, to like even the side by side learning this education and then side by side actually implementing it in practical life. So I think parents… that would be a huge misconception for parents that you're kind of guiding our kids in the wrong way, even though it's a good thing to do. |
| | | Respondent 11, Female, Teacher Urban, Age 42, Pos. 21 | Interviewee: They would believe that if they educate kids about the sex, they might start practicing it. So, I would say that in that case, they don't believe there's any effectiveness there. |
| | | Respondent 11, Female, Teacher Urban, Age 42, Pos. 26 | Interviewee: There are a lot of misconception. As I said, the very first one is that they believe that educating their own kids, if they have sex education, they probably feel it's appropriate to do at a young age. So, this is the biggest fear, which many parents think that probably they should not talk about anything like that. And I believe it's a myth, because if they don't know, anyone can take advantage of the ignorance. |
| | | Respondent 13, Male, Doctor, Age 35, Pos. 84 | Interviewee: Stigma while reality, it makes the innocent even more aware and tend to instigate and provoke them to the things, which are out of their bounds. Make them curious, make them tend to hyperactive their hormones, tend to get from the right part and adopt a part of going towards hell and their youth is ruined and whatnot and all, they can bring up so many things. |
| | | Respondent 15, Female, Health Department, Age 31, Pos. 84 | Interviewee: The biggest misconception is they feel that if their child would be educated regarding these things, they would … it would negatively impact their personality in some way. |
| | | Respondent 18, Female, Teacher Urban, Age 30, Pos. 66 | Interviewee: Yes, there are. I think that a lot of them stem from the fact that these children are sort of rebelling against your local customs, your local traditions. They're rebelling against Islamic values. There's less of an understanding about the fact that it is a physiological change, that it's a hormonal change and that it's a challenge itself for children to maneuver and go through it and navigate their life through it. Most of their behavior is then just put on as being ungrateful or in some contrast to societal cultures and norms and religions, and then that becomes an issue. People, instead of actually understanding them, will push them aside and will consider the issues that they're actually facing irrelevant. |
| | | Respondent 2, Male, Teacher Urban, Age 29, Pos. 100-102 | Interviewee: And any kind of education about contraceptives or any kind of safe sex practices, they think that that's just going to make it more easy or make people more likely to have engaged in sexual activity before they get married. ……They believe that the education about these things is going to lead people to having more sex, which is what they think is going to move society towards a lack of morality. |
| | | Respondent 21, Female, Parent, Age 35, Pos. 54 | Interviewee: I think so, there are misconceptions. That's the reason why it's not being taken up at a national level and it's not being implemented in school. That is self-evident. |
| | | Respondent 22, Male, Parent, Age 35, Pos. 46 | Interviewee: A lot. First of all, they don't really have an idea about what sexual education or sex education exactly is. It involves a lot of things apart from like this, maybe prevention of diseases, sexual health, mental well-being sexual well-being. They don't understand that all of this is included in that. What they really understand out of sex education is to teach children about what sex is and how to maybe practice safe sex. They don't really have an idea about that it's much more than all of this, and a lot of things need to be addressed. Diseases need to be addressed. As mentioned earlier, the mental well-being, the sexual well-being all of that needs to be addressed. |
| | 2.5. Dangerous consequences | Respondent 11, Female, Teacher Urban, Age 42, Pos. 28 | Interviewee: We have several incidences…..in Islamabad.….people tried to attack them with stones and rocks and so in Islamabad there's a huge group of people particularly which are very conservative..... They don't have any acceptance for this kind of knowledge or education being done. |
| | | Respondent 11, Female, Teacher Urban, Age 42, Pos. 69-70 | Oh, there could be a lot of things showing the opposition in terms of everything coming in the newspaper, even in journal, people posting stuff on, saying things against any particular group who's doing it, who's supporting it, they can be things in the newspaper, in the magazines. It's on social media. And there might be people protesting against them. They might be people, a group of people who are very loyal and might try to harm those people. So, when it comes to opposing, that can come in any form. |
| | | Respondent 13, Male, Doctor, Age 35, Pos. 66 | I mean God, people talk America is the most liberal society, where you can voice out opinion. No, you don't know the power of clergy in Pakistan. They can block the road, they can formulate this issue with blasphemy, for their interest, they can manipulate and extrapolate things to a very hazardous level, in a very toxic manner. They can block the roads, they can start violence and agitation. … This is not the way that we were being brought up by our parents and forefathers and sisters and this and that. So definitely they can start agitation, there can be lockdown and violent protests. |
| | | Respondent 32, Male, Doctor, Age 40, Pos. 62 | And my own topic of research in the community while in medical college was about contraceptive use. It's a little off topic, but I'm just giving you an idea that just the contraceptive use, when we went to the different villages and we used to ask people about use of contraceptives, we were kicked out, we were beaten up, our female members were slapped. So contraception is directed towards adults and talking about contraception met with so much criticism. So just imagine that talking about sexual issues with a minor, what do you expect? |
| | | Respondent 7, Female, NGO Employee, Age 30, Pos. 47-48 | For example, when they were talking about just say reproductive rights at the Aurat (women's) March, they started throwing stones and sticks. It can range from verbal abuse to physical violence through, like, active protests. |
| | 2.6. Dissemination of efforts | Respondent 10, Male, Political Activist, Age 30, Pos. 18 | Even though they might not know exactly which NGO or which body or which group is performing these activities, they will have a general idea about this. |
| | | Respondent 24, Female, Health Department, Age 32, Pos. 21-22 | Interviewer: You said it's going to be disseminated, and are there any obstacles in implementing this project? You can think of any obstacles or barriers?<br>Interviewee: Yeah, up till now, because it's within a ministry, we are not facing any challenges. But yeah, once it is disseminated, it's 100% sure that we are going to get challenges or obstacles from the religious extremities. And I think Pakistan covers 30% to 40% of religious extremist idea. |
| | | Respondent 24, Female, Health Department, Age 32, Pos. 82-84 | Interviewee: Not brochures and pamphlets. They are not available…… But yeah, reports are available on this.<br>Interviewer: And do the community members access to this information? Do they usually use this information?<br>Interviewee: Yes, from Islamabad's perspective, 2% of the population. |
| | | Respondent 3, Female, NGO Employee, Age 27, Pos. 90 | Interviewee: Well, it depends in what way the efforts are being made to address this issue. So if it's some kind of a charity organization, I think they may not necessarily get a lot of opposition, because mostly their work is very unrecognized. It's not brought to the limelight generally, but if it were like a movement or something, that's more, you know, mainstream in the media, they might show through opposition through the media. |
| | | Respondent 4, Female, Teacher Urban, Age 24, Pos. 41 | Interviewee: Yes, there are good efforts, but people should have access to it. |
| | | Respondent 5, Female, Teacher Rural, Age 23, Pos. 17-18 | Interviewee: I don't think they can name any hygiene sanitation efforts, but they can name every other kind of effort that has anything to do with finances. Other than that, they don't really care. |
| | 2.7. Disconnect with the community | Respondent 10, Male, Political Activist, Age 30, Pos. 43 | I'd start with weaknesses. I'd say one of the weaknesses that they're often not aware of the material context of these people. So, I think, for a lot of these programs, relevant to these people's particular material condition. Often times, it's hard for maybe people to relate to what they're listening to. That is one weakness. |
| | | Respondent 8, Female, Teacher Rural, Age 23, Pos. 30 | Interviewee: Because they feel a lot of the efforts that have happened, they've been very removed from the people themselves…….So even the people that were dressed in, you know, jeans and they're talking in a language that's not very accessible to the students or whoever is listening. |
| | | Respondent 8, Female, Teacher Rural, Age 23, Pos. 32 | Interviewee: I think that they don't have enough of an understanding, because, so this is a sensitive issue for a lot of communities and to get through to people is something that's, you know, more engaging than one seminar on it. And like, you know, you need to have conversations and you need to build that sort of purpose in the kids, whoever you're addressing even when they have sessions for sometimes about pregnancy or something like that. |
| | | Respondent 8, Female, Teacher Rural, Age 23, Pos. 36 | Interviewee: Yeah, there are some women as there is, for example, seminar, the health related issues for women. So even the problem is people don't start from the basics. |
| | 2.8. Western concept | Respondent 32, Male, Doctor, Age 40, Pos. 49 | So I think that the majority, the majority opposition that will get is from conservative organizations and they are very much against anything that is remotely westernized. And since sexual health and sexual awareness is a westernized concept. So I don't think it will be taken to very kindly and they'll come out on the streets, they will oppose them. |
| | | Respondent 4, Female, Teacher Urban, Age 24, Pos. 97-98 | Interviewee: Yeah. It's for the same reasons that religious concerns and the cultural concerns, it's a westernized concept taking over. So no, I wouldn't say a huge population in Islamabad, but there is a minority, is a sizable part. |
| | | Respondent 5, Female, Teacher Rural, Age 23, Pos. 109 | Interviewee: Okay. There are a lot of students of mine who have never had polio drops because their parents think that it's some kind of an agenda, and it's not good for you, and it's going to cause reproductive problems. We recently had a deworming campaign at school, and a lot of parents called us beforehand and told us not to let their kids have the vaccination. They look at this as a Western agenda that is sent just to make their kids more liberal and more outspoken. That's just the community perception about this whole thing. They're not open to anything that they feel like will threaten their influence on their kids. That's it. |
| | 3.1. Strategies for implementation | Respondent 1, Female, NGO Employee, Age 27, Pos. 37 | Interviewee: Yes. Yes. If you could ask them, because as I told you, the three tier system in Islamabad, so if you're able to ask them, because they meet with parents, you know, they have those PTM's, parent teacher conferences, PTC's, so they discuss… they have this mentality of how parents and how students would take this topic. So I think if this could be discussed with them and then their views and concerns could be added to the study, it would be very helpful. |
| | | Respondent 12, Female, Health Department, Age 36, Pos. 119 | I think the first strategy is to advocate this issue along with the evidence. I think the parents are… the easiest most like target would be the parents, because already this is an area of great concern for parents. |
| | | Respondent 2, Male, Teacher Urban, Age 29, Pos. 163 | I mean, in the ideal case, it's quite possible. I mean, the ideal case would be that if the religious leadership also got involved in this. On the weekly Friday sermons that they are having in the mosque, they could talk about those things. But the way they usually talk about these things is never really hopeful. So that would be the ideal case, because that's going to get rid of the taboos associated with sex. |
| | | Respondent 2, Male, Teacher Urban, Age 29, Pos. 166 | So the only reason I talked about getting the religious aspect on… the religious parties on board is, that if they are not taken aboard, if they are not brought alongside these kinds of decision making, then sex education is always going to be doubted. And there's always going to be some kind of resistance with the sex education. So the only way you can have a long lasting change is that either you get rid of the backlash that you can get, or you bring them on board. Otherwise there's always going to be some kind of a backlash coming from that side of the community. |
| | | Respondent 20, Male, News Reporter, Age 30, Pos. 110 | Interviewee: The number one thing which I see is access to information and probably advocacy so that people get to know about what actually the problem is, and what could be done to solve it. Once people get to understand this, that what are they actually facing and how actually through collective community efforts it could be solved, certainly that would be a good thing to start off from. That leads towards a better solution to the problems. |
| | | Respondent 20, Male, News Reporter, Age 30, Pos. 112 | Interviewee: Generally, what I was telling you about it, specifically about it, I guess as this very area is concerned to child education, so probably one way could be that we could consider to increase outreach of information to parents and teachers and other stakeholders as well. Probably authorities who regulate the education sector or… Then if we can actually use different forums like… What I'd ideally like would be to focus on this very idea, notion of passing information in a more scientific way so that we have very good data for our target audience. For that, we can do a lot of things to actually see what very segment of the community should we actually target for such information campaign, say. |
| | | Respondent 33, Female, Head of School, Age 30, Pos. 84 | Interviewee: I think one of the strategies might be to start with the more prevalent consequences of the problem, rather than starting with identifying the problem, identify the consequences and talk more about how this problem is right now manifesting itself in the community. And then create a path to the original problems that can be one way of going about it. |

| | | | |
|---|---|---|---|
| | | Respondent 35, Male, NGO Employee, Age 51, Pos. 106 | There should be some pilot project, but somebody needs to design that project. You give them the context of Pakistan, so people accept this project. If you design it with the context of Europe or America or Germany or Italy, it will not be successful in Pakistan. You need to design a project with the context of Pakistan society. |
| | | Respondent 1, Female, NGO Employee, Age 27, Pos. 23 | Leaders, some leaders may show their support and some not. It depends on what kind of political organization or party they're related to, but in Islamabad, there are a lot of organizations that are working towards reproductive health, but that's more on the abortion side or the gynecology side. It's not related to sexual reproductive health concerning girls. So I would say that still the government is not giving them the NOC (Non objection certificate), which is the no objection certificate to work in the country. So they're working just because they're related to an international organization, but I would say that if they were able to say yes to such organizations, it would have a huge impact on the population that is to be studied under this topic. |
| | | Respondent 10, Male, Political Activist, Age 30, Pos. 46 | Besides what I've already mentioned, so, NGOs embarks on these days are having a very hard time. They're actually closing up shop, and a lot of them are actually not working anymore. But the government does have some efforts, but even then, I mean the government's only effort is basically regarding contraceptives and family planning through lady health workers. |
| | | Respondent 31, Male, Political Activist, Age 31, Pos. 94-95 | Interviewee: Probably, if you talk about the civil society or the small NGO's working for such causes, they will be doing that, but there will be just, you know, there will be taking grant approvals from the international organization or such, you know, institutions who are working in this domain. |
| | **3.2. NGOs** | Respondent 35, Male, NGO Employee, Age 51, Pos. 72 | I think by the private sector or NGO's, not by the government (current efforts). |
| | | Respondent 4, Female, Teacher Urban, Age 24, Pos. 121-122 | Interviewer: How are current efforts funded by government, NGO's, private sector.<br>Interviewee: NGO's and private sector. |
| | | Respondent 5, Female, Teacher Rural, Age 23, Pos. 8 | Interviewee: Yes. There is an NGO called Rozan. They have these little kits that they distribute in schools, and they also have little handbooks about the basic menstruation things that the girls should be aware of. Actually, when we were getting trained for our jobs as well, they taught us that the highest reason for female dropout in Pakistan is the fact that the bathrooms are disgusting and the girls have absolutely no idea how to deal with this. Rozan is one NGO that specifically works for this, about sexual harassment and about female hygiene. They have really nice instruction manuals and things as well. |
| | | Respondent 5, Female, Teacher Rural, Age 23, Pos. 45 | Interviewee: Yes. Everything that we do is evaluated, especially if it's going to be… Any NGO that works with sanitation and health, even if it's a big corporation that's just trying to reach out for that… Everything has an accountability. There are surveys conducted. There are phone calls conducted with the parents. There are interviews. There are several different forms that are filled out. I know this because even we do it and everything is tracked. Then they'll see the content, how many people showed up, how many people did not show up. There's a very strong follow-up chain that goes in after every kind of action like this. Then all of that feedback is incorporated to make this content better and more approachable. |
| | | Respondent 1, Female, NGO Employee, Age 27, Pos. 144 | So I would say that nowadays media, social media and internet are the two most likely platforms, where parents visit, kids visit, everybody's on there. And it's a very huge hub, where people are interacting with each other, sharing their opinions. So I would say that something like more of like a campaign or I don't know… I don't know what the name is, those videos where they have like those cartoon characters, you could say, like they have characters, main characters in which they show a message through that video. |
| | | Respondent 1, Female, NGO Employee, Age 27, Pos. 145 | I would say something like that that could be highlighted and given importance. So once that's shared, when it's shared once, imagine it being shared a million times all over the internet, all over social media. And I would say that forwarding messages, such as in your phone, like WhatsApp and other message hubs, they're a source of connection to parents. So something that is being liked and shared on WhatsApp and it's being forwarded the whole, you know, family or connections or friends. It's a huge you could say platform, where something like this can be shared |
| | | Respondent 1, Female, NGO Employee, Age 27, Pos. 149 | So I think the TV is a very good source, where they could… and everybody now I bet has access to a television at their home. So something that could be locally or locally, like given through a message, like a video maybe, like a one minute or two minute video, where this kind of topic is being discussed. Or I would say Pakistani, especially mothers, since they're so worried about their children, they're very interested in like TV series, different shows in such of that. So I think if those kinds of things could be brought into locally spoken serials, you know, like series in which this topic is being discussed. |
| | | Respondent 1, Female, NGO Employee, Age 27, Pos. 150 | Because when one kind of series or a show or something like is hit, every mother, every woman in the community is watching that and they're talking about it and they're gossiping about it. So it's like they're sharing their perceptions about it. So, I mean, I think that would be a good platform where this kind of idea can be brought into. |
| | | Respondent 17, Male, Parent, Age 29, Pos. 7 | Interviewee: So, I manage a startup incubation center in Islamabad, where I'm working directly with a lot of health-related startups. I've worked with a startup called Sehat Kahani and Augment Care. And there's another one called Web Respondent. And all three of these startups are working in the telehealth domain. And what they have is they've got a pool of Doctors available online, that you can speak to with complete confidentiality. And now, this trend is actually picking up in the urban centers of Pakistan. |
| | **3.3. Digital media, social media, and online platforms** | Respondent 17, Male, Parent, Age 29, Pos. 79 | Interviewee: I think the most important thing is to essentially use the state television to actually create support around this issue. And the reason for that is that state television over the years is known to be a conservative channel. A lot of people have a lot of trust on the state television and they feel that a lot of information that comes on it represents our culture and our values and our value system. |
| | | Respondent 2, Male, Teacher Urban, Age 29, Pos. 159 | I think they should follow the strategy of Greenstar, which is a contraceptive brand here. As a kid, I remember their ads being all over the TV, where they were talking about condoms and other forms of contraceptives, not directly, but they were talking about Greenstar and it was more the families. So if actually mentioned that these were contraceptives or they were talking about sex related things, they were just saying Greenstar small family. And in that way, they were able to make a place in this society. So something of that sort, where they are talking about it, but not directly, |
| | | Respondent 21, Female, Parent, Age 35, Pos. 20 | Interviewee: I believe the social media activists. They might support this cause, and when we do have these student council unions. They might support this cause. That's what I believe. |
| | | Respondent 23, Female, Parent, Age 29, Pos. 87 | Interviewee: That is what I mean by media. It's basically social media. It's basically your news. It's the celebrities that would come forward just to provide awareness. For an informed person, I think it holds a responsibility. They'd come up with certain problems in the society and the rest and just because people will listen to you. So, I think yes, social media, Twitter, Facebook, YouTube, everything should be included when it comes to waking awareness. |
| | | Respondent 34, Female, Social Media Influencer, Age 38, Pos. 14-15 | Interviewee: I mean, I've seen people on like… since I've… when I was in Pakistan, I've never seen anyone trying to do that, but since I've moved here and the social media thing is very in and it's easier to get in touch with people and you can do a lot online. So I have… I'm a part of some of the Facebook groups, in which there are… that's like a women only group, where people come in and discuss their problems, their sexual problems, their problems related to birth control or their problems related to their sexual life, married life and stuff like that. So they talk about it and other people kind of help them with it. That's the only thing that I have seen. Otherwise … yes. Like even the people who come up with their problems on those pages, don't come up with their names. They're mostly… because see, there's this taboo that they don't want to tell their names. They are like… they contact the admin of the group and then they come up as an anonymous message and then people give their opinion about it. That's the only one I would… I can think of. |
| | | Respondent 13, Male, Doctor, Age 35, Pos. 136-137 | And coming to the other barrier, where the parents despite the generation gap have a misinterpretation, because they tend to focus more towards… to these mullahs as compared to the worldly education. …… It's just not, since they converse in English that reflects how literate they are, but they have very rational thinking and very clear concept of the Holy Koran and they can correlate with other religions as well. So they, in fact, by such virtual education a lot of misconception has been wiped off and such means of education and literally sermons tend to be very effective in wiping out the misconception from the elderly population. |
| | | Respondent 13, Male, Doctor, Age 35, Pos. 140 | And get some coaching, some seminars or workshops by the such representators, who just tend to write proposals. And I mean, I'm sorry I'm targeting the public health community… they should impart such knowledge and conduct workshops at schools. If the school gives them the incentive of conducting a workshop and pay them a good amount as an encouragement. |
| | **3.4. Community engagement** | Respondent 29, Female, Head of School, Age 29, Pos. 95-97 | Interviewee: I mean, there are just a lot of parents that I know of, who would be like really willing to be a part of different conferences. If we were just, sit down and have like an interactive in session with them and even just talk to them, like perhaps a session dedicated to just this issue. …… It's like a walk on eggshells really. So you don't want to trigger anyone and it's just, yeah, so there are no professionals and they cannot have the talks and then the adults cannot have the talks with the children and it's just a whole vicious cycle. |
| | | Respondent 3, Female, NGO Employee, Age 27, Pos. 166-167 | Interviewee: I think a good step would be for… like you were talking about leaders bringing up the issue, because they have a big… they have a large outreach. ……. And I think also getting parents on board especially in schools if you for example, if you get the parents of the kids on board, I think it would be … maybe you could hold seminars specifically designed for parents, just to explain the importance to them so that you can further design a curriculum that has all of this information. |
| | | Respondent 9, Male, Teacher Rural, Age 23, Pos. 28 | If it's by a community leader who is very influential and it's a part of a political movement, then I think it will gain more traction. |
| **3. Strategies for tackling resistance** | | Respondent 9, Male, Teacher Rural, Age 23, Pos. 36 | The parents I talk to, they tend to open up and talk very frankly with teachers like us who are younger and who tend to be empathetic or try to understand their issues. But they usually don't actively support because of some of their own interests, sometimes because of time constraints, sometimes, resource constraints stop them from actively supporting them. So, passive support is mostly there. |
| | | Respondent 10, Male, Political Activist, Age 30, Pos. 12 | We did a lot of mobilization in these communities for that (sexual abuse/violence). And that is mostly one of the key demands during that was also regarding gender-based violence or sexual-based violence and stuff like that. So, there are definitely people who are working there. I would also say that the people there are receptive to it. They're not immediately hostile as you would expect them to be. But again, it comes very low in their list of priorities. |
| | | Respondent 13, Male, Doctor, Age 35, Pos. 8 | He (political leader) made sure that the child abuse is a criminal offense now. |
| | **3.5. Sexual violence** | Respondent 15, Female, Health Department, Age 31, Pos. 13-14 | Interviewer: Okay. And are they specifically working on sexuality education?<br>Interviewee: Not that I think they're working more on violence against women and rape, but I don't think on sex education, no, I don't think anyone is working on that. |
| | | Respondent 3, Female, NGO Employee, Age 27, Pos. 20 | Interviewee: So, there's this community service program that everybody in the university has to go through. So some schools suggested that they go to schools in the squatter settlement communities and talk to the children over there about sexual harassment and just generally how to take care of themselves against sexual violence. |
| | | Respondent 9, Male, Teacher Rural, Age 23, Pos. 22 | The parents I have talked to, they are very much concerned about the physical safety of their own children, and they want them to be safe from abuse, they want them to be safe from violence of any nature. So, if there is something, some structured way of teaching these things, I think most of the parents will be receptive of this idea. Excluding a few of them. But in terms of outside parents, I can't say much because they are religious community members, there are some community members who haven't gone to school or are not very literate. |
| | | Respondent 10, Male, Political Activist, Age 30, Pos. 130 | It is absolutely necessary to get the religious leaders on board. And I think, again, once you build that trust with the community that you are serving this community, once they know that you are doing the community a service, these communities are very distrustful of the government because they had only been exploited by everyone in the past. Usually, their story is one of exploitation. And so, they're very distrustful of any such intervention. So, if you bring them, if you grant them a trust, if you basically address their issues, I think they will be more than receptive to anything you do afterwards. And anyone who opposes this in the larger community, whatever they say will have that much weight because the community at large will trust you more. So, I think it's necessary to address them because they gained obviously a lot of social capital. And I think it's entirely possible to do that once you build that trust. |
| | | Respondent 13, Male, Doctor, Age 35, Pos. 134 | The education of clergy or mullahs and mullahnas is very important. Unfortunately, in our society, the dilemma is when we hire the leader for … to run the congregational prayers or head of the mosque, we just see how much road land he has, how much road learn he has, the Holy Qur'an by heart. We don't know what is his intellectual level, they have zero intellectual level. |
| | **3.6. Opportunities of awareness** | Respondent 15, Female, Health Department, Age 31, Pos. 133 | Interviewee: I think what's important is that the religious leaders also need to understand that it's a very important aspect of the religion as well. And there's nothing wrong or bad about it, if it helps to stop the greater evil. And I think once you can get the religious leaders on board, then you can also get the political leaders on board. |
| | | Respondent 15, Female, Health Department, Age 31, Pos. 139 | Interviewee: So once you get the younger lot on board, even if you don't get the older ones, you still have a significant percentage of religious leaders who would support you. |
| | | Respondent 15, Female, Health Department, Age 31, Pos. 143-144 | Interviewee: I think television would do good, because I remember we used to have these ads for HIV AIDS, when the outbreak took place in Pakistan regarding its awareness......So we can do that for sex ed as well. I think that should reach, because I think television is what almost every household has at least one set........ So I feel like if we use say the newspapers or the television that should have a greater impact than if you could just go to schools and kind of aware of them and aware the kids regarding that, because we used to get like certain awareness sessions when we were in high school that used to help us. Yeah. |
| | | Respondent 3, Female, NGO Employee, Age 27, Pos. 92 | Interviewee: Yes. I mean these issues have great consequences when people, you know, when you shine a light on those consequences, I think people would actually understand why there's… why addressing these issues is so important. |

| | | | |
|---|---|---|---|
| | | Respondent 3, Female, NGO Employee, Age 27, Pos. 94 | Interviewee: Yeah, yeah. Because if you like… like I said, there's a very negative connotation attached to sex education. So if you basically address the reason why it's so important and target, you know, bring out why the consequences of this can be so terrible, I think people will be more open to the idea. |
| | 3.7. Age appropriateness | Respondent 14, Male, Teacher Rural, Age 23, Pos. 52-53 | Interviewer: How many community members know regarding age appropriateness of adolescent health syllabus, like it's an incremental approach.<br>Interviewee: No, I would say not at all because it's all a taboo topic, which people don't go near that. |
| | | Respondent 19, Male, Parent, Age 32, Pos. 48-49 | Interviewer: And regarding age appropriateness that the contents are usually age-appropriate. How many would the community members would be doing?<br>Interviewee: Not at all, I guess, because they don't know exactly what to tell. |
| | | Respondent 20, Male, News Reporter, Age 30, Pos. 71-72 | Interviewee: No. They would not be knowing about the concept of age appropriation, I guess, most of them. |
| | | Respondent 24, Female, Health Department, Age 32, Pos. 121 | Interviewee: No, I had just checked your query about age and you told me you cleared that CSE is age-appropriate. So, I think if you are going to add this initiative in the curriculum for children, so this should be in the mind that, for example, in 5th grade, that would be the basic things we are going to tell them and then 6, 7, 8 according to the grades. |
| | | Respondent 28, Female, Health Department, Age 26, Pos. 59 | Interviewee: So yes, one is as you said earlier that one is that they might not know about the age appropriate content, so they might go like it is like ruining their child's mind by telling them everything. Then secondly, the misconception is that if you inform the children about it, they might think to have a more … have an increase in interest towards it. So probably that is one of the barriers for providing them with the education. |
| | | Respondent 3, Female, NGO Employee, Age 27, Pos. 175 | Interviewee: I really think that it would be very crucial that this is… you were talking about incremental education, I think that's very important, because there's assets, as far as I know. And even at this point in the education system at the earliest levels or even the in high school or university, this is not something that's talked about at all. Even in the most expensive private schools, it's not talked about, where you would expect that people are more open to the idea of such an education curriculum, but it's never talked about. |
| | 3.8. Masking or optimizing the CSE | Respondent 14, Male, Teacher Rural, Age 23, Pos. 26 | Interviewee: By vocabulary (not to use/change), I mean, the kind of words that would be perceived as not very controversial. So, if they would have like word sex, sexual harassment, that would help them get the topic across without saying something that would not be listened to by the community. |
| | | Respondent 4, Female, Teacher Urban, Age 24, Pos. 146-147 | Interviewee: You're going to think I'm a very bad person for this, but I honestly think we need to cover it up with something else. We need to say that this thing does this thing that they want and then actually do whatever the hell we're doing. Like it's… there's no other way there. You have to lie about it, in like… in a very like diplomatic way. Like, you know, like a blanket statement……like saying that this is specifically to tackle sexual abuse in children, for example. And then show like you know contraceptive techniques or like safe sex techniques or hygiene techniques or how to handle a pinprick situation, which a lot of Doctors don't even know, you know, things like that. So like I feel like we need to lie a little, like I genuinely think, because it's, it's better PR, it's like, that's the only way that we're not going to get resistance. You get these … these forces are very powerful. Our opposing forces are very powerful. They're very idolized in our country and our culture and this is a terrible solution, but this is solution that I see. |
| | | Respondent 4, Female, Teacher Urban, Age 24, Pos. 149 | Interviewee: Okay. So I feel like we need to sell it. We do like sell this idea of like sexual health to specifically our religious leaders. And by that I think that we need to skip some things that we're talking about in this curriculum, especially when it comes to adolescent health. Right. If we talk about when we're convincing them that this is something worth doing. |
| | | Respondent 4, Female, Teacher Urban, Age 24, Pos. 151 | Interviewee: Yeah. So to make them see that it's worth their while and by omitting a lot of the stuff that we will be including in the curriculum and then doing it. That's what I think is a solution by lying by omission, because that's the only way we're not going to get a backlash for it. I don't know. Like I just want to bypass the religious like stuff here. I'm not a lawyer, if you can't tell, I don't have a lot of information about this. |
| | | Respondent 4, Female, Teacher Urban, Age 24, Pos. 153-154 | Interviewee: I think as an individual, it's very easy to do this. If you're not like affiliated with a big organization, it can work in your favor and it cannot work in your favor. Individualistically a lot of artists do try to integrate this stuff, if they're teaching to their kids. It's very important to know how to build a safe space. And a parent can do that, a teacher can do that, a principal can do that, a Doctor can do that too. In every area, if, as an expert in your field, if you are shown how to create a safe space and that means like changes in your language. |
| | | Respondent 7, Female, NGO Employee, Age 30, Pos. 118 | Interviewee: I'm just going to summarize what I said before. I feel like it just needs to be sold to them in a way where they feel like it's personal and it's beneficial for them, and it's not a Western idea or an outsider concept that's not relevant to them. It has to be adapted in a way and presented to them and to everyone to get them on board in a way where it feels personal and very relevant to them. |
| | 3.9. Peer to peer or parent to child | Respondent 14, Male, Teacher Rural, Age 23, Pos. 55 | Interviewee: I think some of the biggest misconceptions that I have seen is the whole topic of sexual education, it's not something that they think might benefit their students because they go through the very traditional roots of sexual education. And that is either peer to peer learning between students, or parent to children learning near the age of marriage or sexual maturity. So, yeah, I don't think that's going to be a lot. |
| | | Respondent 18, Female, Teacher Urban, Age 30, Pos. 60-61 | Interviewee: I think that that is where the conversation should personally start (schools). What it does is it allows children of the same age, similar demographics to all experience the topic together. They can sort of learn together. There are little to no social taboos attached to discussing with your peers about something. We know that children generally are more open and more receptive to whatever it is their peers are doing. |
| | | Respondent 18, Female, Teacher Urban, Age 30, Pos. 62 | If you tend to make something trendy or fashionable or okay for a particular age group, then children tend to respond to it more. As compared to children talking about a particular thing, but unable to discuss it with their parents or their grandparents or their teachers. When this discussion comes from school, you essentially have the whole trifecta of parent, teacher and your friends all on board the same particular thing, so it's easier to discuss it and it's easier for children to be more open to it as well. |
| | | Respondent 32, Male, Doctor, Age 40, Pos. 2 | So they would rather want to, you know, like skirt from the issue and let the children learn from themselves from other sources. And that is actually harmful, because peers can give you very wrong information, misinformation and that would lead to the development of a very poor and very skewed sexual health and sexual identity. |
| | 3.10. PSHE | Respondent 18, Female, Teacher Urban, Age 30, Pos. 5 | Keeping this particular fact in mind, private schools have recently started introducing different modules that cover health education, sex education as well. I used to teach pre-O levels in a private school, in Froebel's and they had recently implemented a subject called PSHE, Personal Social Health Education. I believe it's also compulsory in the UK. They had brought actual people who had background in development to help them develop a curriculum for it. There was a very big readiness for it, and they wanted to introduce it to children from grade six onwards, which essentially when you're 11-12 years old. I saw that happening at private schools where a discussion, led by teachers, was initiated for this purpose. |
| | | Respondent 4, Female, Teacher Urban, Age 24, Pos. 8 | But now in schools, especially head start, beacon house, they had started something called PSHE ……. In it through a very comprehensive and holistic approach, they are able to talk about where the boundaries of me begin and end, what our relationships are and what our boundaries within relationships. What is a good touch, what is a not good touch, how do you respond to strangers. How do you… about even power dynamics, like how power dynamics shift from poor rich or older to younger, like things like that. |
| | | Respondent 4, Female, Teacher Urban, Age 24, Pos. 10 | So there is an approach and luckily PSEG starts from a very young age. It starts from kindergarten to all the way, to all levels. In head start, what we do is that in most branches of Islamabad, it has started it… it functions on the IB curriculum, which is also a very holistic syllabus and curriculum. It depends on personal and social development specifically with regards to sexual health and stuff. |
| | | Respondent 4, Female, Teacher Urban, Age 24, Pos. 13 | Interviewee: I believe PSEH has been in action for three years at the very least, if not more. And I've only just joined head start, so I can't really say how long it's been happening. I don't know how long it's been fully functional. |
| | | Respondent 4, Female, Teacher Urban, Age 24, Pos. 35 | Interviewee: So I feel like I'm being very pessimistic in this interview. Mostly like it's just a lot of paperwork, on the surface of it, it is a lot more paperwork. You have a specific PSHE teacher that means, you know, there's one more job, that means there's more papers required, you know, sort of keep portfolios and all of that. For teachers, I would definitely say that even for students and when you have new curricular, you need to have trained personnel as well. |
| | 4.1. Leadership | Respondent 1, Female, NGO Employee, Age 27, Pos. 34-35 | Interviewer: Okay. Who are leaders that are supportive of addressing this issue in the community?<br>Interviewee: Leaders would I think be the governors of the city. So anybody that's in tight niche with the government schools, especially leaders, the people that are the leaders, basically the people that are working in the educational level system, who are taking you know, in close proximity there, they know about the schools and everything about the… I think if they know the leaders, who know about… who are part of the educational department, those are the leaders. Yes, those are the leaders that should be in charge and should know and should be asked if this kind of subject should be implemented in schools, in colleges. |
| | | Respondent 10, Male, Political Activist, Age 30, Pos. 53 | Yes. The leadership does participate, and I'm talking about local leadership. So, they do participate, but their participation is not necessarily because about what they think of these efforts. It's usually tied to their role within the community themselves and their role as basically patrons that act as a liaison between these NGOs or these organizations, these political parties, and the rest of the community. So, for them, it's just a way to exercise or increase their influence. They're not that much concerned with what exactly the nature of the intervention is, but they are receptive to it. |
| | | Respondent 10, Male, Political Activist, Age 30, Pos. 63 | Interviewee: How it works is that basically a lot of the people who are working with these organizations have some political standing within these communities. So, they do count, even if they don't wield any direct political power that they're not any part of, for example, local bodies, even if they're not part of the local government, they still will somebody power in that that they have control over the votes in that area. So, they can be called, in a way, political leaders and these are the ones who are mostly leading these efforts or organizing these efforts rather. And so, yeah, I think that would be the primary leadership. |
| | | Respondent 11, Female, Teacher Urban, Age 42, Pos. 51 | Interviewee: I'd say female leaders would support it more. If we have females in any, then males. Because females probably they understand that they might have seen that happening in their own families, they might have experienced it themselves. Because there's so many untold stories. So, I believe in general, my opinion towards this will be females will be more supportive than males. |
| | | Respondent 11, Female, Teacher Urban, Age 42, Pos. 53 | The only type of leaders that I think might oppose would be anybody who's like very religious, because we do have, in our politics one or two religious parties also there. So, I think those are the ones that I believe would be, because they are against even a female liberation and there are so many things. Well, how can they allow? Because once we talk about educating humanity, it's not just males, it's females as well. So, those kinds of leaders, I believe are going to highly and strongly oppose it, and then that can be followed by a few others. |
| | | Respondent 13, Male, Doctor, Age 35, Pos. 36 | I think considering this issue right from the individual messes to everyone in the aristocracy would definitely support it. It's just not a minister for interior would say, sorry, it's not my office. Obviously not. Being human in nature indefinitely raise his voice for the defense of the children or would definitely want such a pain to be inculcated in our curriculum, especially who are actually working proactively for the curriculum standards, being the education minister. But yes, everyone would be vocal in putting up the stands forward, but just vocally, no one would be physically active. |
| | | Respondent 18, Female, Teacher Urban, Age 30, Pos. 31 | Interviewee: No. Not really, that I think of. I think they're (leadership) politically correct in a lot of places, but they don't really like to get their hands dirty. |
| | | Respondent 22, Male, Parent, Age 35, Pos. 18 | Interviewee: I think with the new generation coming in, if the new bureaucrats are approached, they might be open to the idea and they might be in a position to implement that, because if we consider politics, politicians, they're not open to that, they're not approachable. And I don't think they're going to be open to that because a lot of their voting depends on how much they satisfy the religious groups and the culturally appropriate groups. However, bureaucracy is independent of that. They don't really need support from the common people. So, I think if they are approached, this issue might be addressed a little better and public can be approached that way. |
| | | Respondent 28, Female, Health Department, Age 26, Pos. 21 | Interviewee: So as I mentioned earlier, I feel really glad to say that this agenda is being taken up to the parliament as well. Apart from that, minister of … I guess the minister of health and secretary, as well as the following tiers, there is a whole dedicated strategic area of RMNCH in the health ministry that works and is making quite some effort for his particular purpose. Apart from that the, as I mentioned earlier, other organizations, that work in close proximity with government are also putting in a lot, many efforts to push this agenda through. |
| | | Respondent 32, Male, Doctor, Age 40, Pos. 13-15 | Interviewer: Yeah. So does the leadership usually take part in such… promoting such ideas or concepts? Do they take part in such activities?<br>Interviewee: No, they don't. And the reason is… the reason is that they have to, they have to make sure that they're, you know, they're, the votes are coming. And a lot of their votes come from the people who are following. I'm very sorry to use such word, but they were following the religious scholars and so called mullahs. They are following them blindly. So those masters are there that they make up the majority and might lose votes. So they would rather avoid being a part of such a, you know, like a plan or such a you know even idea, a proposition, they would, they would back out. They would take it, don't take our name in it, because if their name comes up in it and they would be associated with the devil or somebody who supports the Western culture. |
| | | Respondent 10, Male, Political Activist, Age 30, Pos. 9 | Yes. There are numerous people who are, I mean, they're obviously NGOs and as political activists as well, who are working with these communities to raise awareness about these issues, especially sexual-based violence, and gender-based violence. So, NGOs like Rozan, for example. They are working in these communities. And, obviously, also, they do have records with the government programs who come into these communities and advice regarding contraceptives and stuff like that. |

| | | | |
|---|---|---|---|
| 4. Current efforts, role of leadership, and policy implications | 4.2. Current efforts | Respondent 11, Female, Teacher Urban, Age 42, Pos. 9 | Interviewee: Those efforts probably might be several NGOs which are making YouTube videos and posting them online. So, that's just giving the awareness, particularly, for females, what are their rights, number one, and what kind of things are appropriate and what kinds of things are inappropriate. But that's just like few NGOs might be doing. I don't know the names of any but I have come across one or two videos online. |
| | | Respondent 14, Male, Teacher Rural, Age 23, Pos. 6 | There is this society, in my community that works on welfare. But I know that there are other NGOs working in neighboring communities that talk about this topic. For example, there's an NGO called Rozan, I think that works for child sexual abuse, |
| | | Respondent 18, Female, Teacher Urban, Age 30, Pos. 17 | Interviewee: In Islamabad I think it's either the education sector or it's the NGO sector. We have a very thriving NGO sector in Islamabad. There are lots of NGOs that are large scale, and then there are also small scale NGOs that are working. As well as there's also a big transgender issue in Islamabad. There's a big community. There's a lot of work being done with them. They're very active on the media. They're very active on social media. I think interacting with those, especially that community interacting with the students has really pushed the direction in a way of discussing issues such as this. I'm positive about this. |
| | | Respondent 24, Female, Health Department, Age 32, Pos. 8 | Interviewee: As I'm working in Ministry of Health, recently, there is a project that has been approved between I think that is a USAID supported project that is a sexual reproductive health. It's not specifically adolescent reproductive health, but adolescent, sexual reproductive health is a component of that project and of the component. So, we are working on that. And it's a two-year project and up till now, inclusion of sexual health in the curricula is not added in that project. But this is the one kind of initiative that has been taking place. |
| | | Respondent 26, Female, Education Department, Age 59, Pos. 9 | And NGOs, most of them are too focused on gender rights and do focus on harassment.........They are trying to give this education in their own way in the community but the teachers are not doing it. Maybe in the private schools in on their own, but not officially. |
| | | Respondent 28, Female, Health Department, Age 26, Pos. 13-15 | Interviewee: I would reach at seven on the basis of like my personal experience working for SRHR, sexual and reproductive health right now. I would give you a bit of context in terms of activities that have been carried out at federal ministry, as well as the cooperation with stakeholders, like nonprofit organizations international, as well as national, including WHO, UNICEF, UNFPA. So, and from the government and the national ministry of national… of health and regulation, as well as the population ministry. So, efforts… right now efforts are being made to incorporate sexual and reproductive health into the universal health coverage benefit package that is going to be launched real soon. The background work for the development of UFC has been done and now it is almost going to get into the implementation phase. So SRHR related activities clearly and explicitly mentioned for the sexual and reproductive health have been added into the package. Apart from this, efforts are being made to incorporate sexual and productive health into the national programs through a dedicated vertical program. It is also being taken to the parliament through the parliamentary secretary for health. Apart from that and other bill is being made, that is for the MNCH, maternal and prenatal response. So that is also a dedicated effort towards, you know, improving the status of health for the woman. These are the few things that are in the pipeline and going to get implemented. |
| | 4.3. Government support | Respondent 10, Male, Political Activist, Age 30, Pos. 98 | The government is doing some funding, but only for that particular to lady health workers essentially, but it's not a lot. That funding is expected to continue, I think, but again, it's not a lot. The NGOs, they usually receive funding from either foreigner donors or the World Bank in development bank or like foreign organizations like that, or to like some interventions by USAID. |
| | | Respondent 10, Male, Political Activist, Age 30, Pos. 99 | That is actually, I think, decreasing because of how it's a bad time for NGOs and banks right now. A lot of them are backing up. So, I think that the money is drying up there. For political parties, the funding it varies quite a lot. So, it will increase as the elections draw near, and then it will decrease, but it varies quite a lot. So, I think on the whole, I think funding around these issues is actually decreasing, and it's going to decrease in the future. |
| | | Respondent 11, Female, Teacher Urban, Age 42, Pos. 48 | Because, see, most of our leaders are well-educated, they understand the importance of this thing. It's just because it's the social stigma, and they have other issues to actually follow. So, it might not be of their concern. But if they believe that another organization is doing it, they might let that happen. Because in the past, all these cases have been highlighted, that Zainab incident and then there was another girl she died in Islamabad in some area. |
| | | Respondent 26, Female, Education Department, Age 59, Pos. 6 | Interviewee: Yes. The NGO sector and the INGOs are definitely working for it and even after the Kasur incidents, the government also said that they will do something, but not much was done. But Sahil and some of the UN agencies and other organizations as well -- one thing which has been implemented in all international NGOs and NGOs is laws against sexual harassment. You have to have a law in all our organizations and it has to be put up on the notice board. |
| | | Respondent 26, Female, Education Department, Age 59, Pos. 33 | Interviewee: Yes. As far as meetings are concerned, as far as if a UN agency will invite, they will participate in the meeting, but implementation doesn't happen, it is just discussion. In discussion purposes, I think if they're invited, they will be happy to be a part of that discussion or that meeting or that conference or that seminar if they're invited as a chief guest but implementation, they should follow that the youth organizations, there are so many I will send you the names, but the architect, once it comes to the implementation to actually reaching the communities to actually getting it done, nothing happens. |
| | | Respondent 28, Female, Health Department, Age 26, Pos. 65 | Interviewee: So yes. If you talk about the government, then yes the government has taken up the initiative of, you know, starting up the sexual and reproductive health program across the nation. And most of the interventions of regarding this will be incorporated into the universal health coverage benefit package. That gives an idea of the sustainability of the project, as well as the implementation of the services. So there is commitment from the political, as well as the healthcare providers. |
| | | Respondent 7, Female, NGO Employee, Age 30, Pos. 15 | When I look at it in terms of, let's say, governance and policymakers, I feel like it's important to them now because maternal and child health is a basic standard now on how a healthcare system is judged in a country. Like, what's your maternal and child… what is it looking like? It's like an indicator almost that judges your health system, your health sector, your livelihood index. I feel like to save face and to show we are making progress, that's why it's of concern. I'm not sure sexual education will be a part of it, but I do know that they want to improve their outcomes and their indicators. If they feel in their head like sexual education will improve this, yes, I think it could be something that would become a higher priority. Overall, I feel like sexual health is a priority for that reason. |
| | 4.4. School heads and teachers | Respondent 10, Male, Political Activist, Age 30, Pos. 69 | But they have very close relationship with these communities. Especially teachers, they recognize this problem and usually at the forefront of organizing for such initiatives, because they deal with both children -- I think that because of their close dealings with children, they're very aware of how this affects them. Women also, I think because they are the primary victims in this because of all of this, because of lack of education around this, so, I think they are also motivated about it. But, generally, the decision-making power and organization power rests with the men who are either dismissive or even if they're accepted to it, it's not because of it's a very pragmatic reason. So, yeah, there's definitely a divide between the people who work in the community and the men. |
| | | Respondent 11, Female, Teacher Urban, Age 42, Pos. 4 | Because, for my own personal experience, I'm a teacher myself, and it's really hard whenever, in biology, particularly any of these topics are coming that has something to do with sexuality, I can always feel the uncomfortable feelings, number one. Number two, I remember my own project I was doing about the cyberbullying, and in that thing, when I talk about sexting, and I was dealing with the middle school kids It was like nobody wanted to say that name and they seems like you know probably I'm all of a sudden started talking something which is highly, highly inappropriate. |
| | | Respondent 11, Female, Teacher Urban, Age 42, Pos. 55 | And again, I believe if I talk about teachers, yes, teachers would be very supportive. But again, I would bring that general aspect into that because the opinions are going to be different. Teachers might accept it or might not accept it. I think parents won't be very supportive. Not all the parents, especially knowing that it's done by a third person or it's done as a group or it might be done at school, they won't be supportive. |
| | | Respondent 12, Female, Health Department, Age 36, Pos. 123 | Interviewee: I think teachers should… training should be given to teacher to sensitize our youth and parents regarding this topic in a very culturally sensitive way. I think they should be given proper training on that. And I think parents… regarding parents, as far as I know, this is like a huge concern already for 50% of the parents, they are really worried about their, you know, young children and their sexual rights and their protection and all that. So for them, I think this should be just advocated nicely that how to, how to tackle with touch issues. |
| | | Respondent 13, Male, Doctor, Age 35, Pos. 82 | It is a stigma, even for the literate teachers, I mean, I remember for my time, when I was studying FSC (high school) and there came the chapter of reproduction and we had a female biology teacher. She asked the guy to teach us the subject. I mean, come on, this is something natural. And she being the female created that barrier, I mean, she could not teach that subject. |
| | | Respondent 14, Male, Teacher Rural, Age 23, Pos. 90 | Interviewee: I think, since I am a teacher and I can talk about my own personal experience, I think I do have a lot of autonomy when it comes to the content that I present in my classroom and how I present it. So, like I said, if there is a curriculum that helps a teacher understand what are the identifiable things that I need to get across, and teachers have that list with them, then it would be easier for them to maneuver around that and design language that fits that immediate detail. |
| | | Respondent 14, Male, Teacher Rural, Age 23, Pos. 91 | And then there could be a glossary of other languages that they could use for these taboo topics. And then community members can hear, I know that there might be a few community members who might be willing to hold seminars about this, who might be willing to hold maybe informal gatherings about this and stuff like that and talk about this. If they have the appropriate language and appropriate visual aids for them, for example, there are brochures and survey and stories really help. If you talk about stories about why this is important. Why we need to do this? What are the benefits of it? Why people need to do it right now, that might be very helpful for the community. |
| | | Respondent 16, Female, Parent, Age 31, Pos. 90 | You need to see and talk to the owners first, who are more inclined toward give them the proper education, who are really not concerned with the wellbeing of the student. … if you really go and just talk a big school chains they will not even entertain you, because they have other things. They are in a race to get into IV leagues, to score higher, to score higher. |
| | 4.5. Policy | Respondent 11, Female, Teacher Urban, Age 42, Pos. 117-118 | Laws might be there. I think, to some extent, in terms of sexual abuse, I think certain laws are being made, but I'm not aware of many. |
| | | Respondent 12, Female, Health Department, Age 36, Pos. 117 | Interviewee: I think that it's about time that these policies should be expanded to address the current need and should not only be expanded, should be implemented, that's the main thing. Because here in Pakistan, we do make policies and they are just, you know, printed on a paper and then left to stay in the cupboard forever. Yeah. So they should be implemented if any such policies do exist. |
| | | Respondent 15, Female, Health Department, Age 31, Pos. 128-129 | Interviewer: Okay. And will it get support by the policy makers?<br>Interviewee: I'm hoping that they… it would get support now, because like I said, there's a very high number of child rape cases. There's a high number of domestic violence. There's a… it's actually the… it's not just the rape cases is not just females, they're actually males as well. So it's the need of time. So I'm I would think that yes, it should be getting support. |
| | | Respondent 7, Female, NGO Employee, Age 30, Pos. 107-109 | I guess it really depends on how you frame it to them and how you're selling it (policies). If you are selling it in terms of, like, "This is going to be better for you as well in the long term…" If you are selling it like, "Sexual education will lead to a healthier community, or healthier women, or healthier people who are running a healthier economy overall in long term sense." Like, "Sexual education equals a healthy woman which equals healthy children, so your offering is now healthy." I feel like if you frame it in that sense, yes, you can get a lot of support. If you frame it in the sense like, "Sexual education will lead to more empowered women who know what to do with their bodies and how to take care of themselves," then I think maybe not. You'll get more backlash. I think it's all about the way you're going to frame it to them. |
| | | Respondent 9, Male, Teacher Rural, Age 23, Pos. 83-84 | In terms of legislation, I can't recall any particular legislation, but I know there is legislation that was passed in National Assembly, which pertains to sexual violence, with children and gender violence with women. So, that's all I know of legislations. |
| | 4.6. Bureaucracy | Respondent 10, Male, Political Activist, Age 30, Pos. 50 | I would divide, if I can, my answer into two parts. One is from the local community leaders and one is from I think the leadership of Islamabad at large. So, I think there is the leadership in Islamabad at large talking about basically the DC (district management) in Islamabad. For them, especially this sexual reproduction education and all of that, that is concern, especially because Islamabad is very densely overpopulated. So, there's always a concern that these initiatives should be undertaken there. |
| | | Respondent 19, Male, Parent, Age 32, Pos. 16 | This quarantined time of this COVID-19, this issue would never have been addressed over here in Pakistan properly, but like leadership is playing a proper role. The DC (Deputy Commissioner) is playing the important role, the other people over here in Islamabad are playing an important role. So, people are actually understanding how a pandemic is working, how transmission is applying, and people are actually taking seriously. So, otherwise, people in Pakistan, at least are very casual about everything. So, even if this issue is put up nicely by the leadership, and I think it can be taken up forward, but it needs to be addressed in the proper way here. |
| | | Respondent 5, Female, Teacher Rural, Age 23, Pos. 34 | Interviewee: For most government schools I feel like if there's a TFP (teach for Pakistan) fellow there or The Citizens Foundation has any kind of link with the school, they will have some kind of an awareness campaign. Other than that, it strongly depends on the area education officer, the AEO. If the AEO is progressive then they reach out to the principals and they want to do better for their school communities. The area officer for our school region, she's new, she's a woman and she's educated. She wants us to even have seminars for the mothers now, so for the next year we're planning on having a lot of that. In most cases, the AEOs are men who don't like to have conversations about this. Unless those communities don't have a TFP fellow or a TCF person involved, there is no way to get in touch with them because we have a huge amount of government schools all over Pakistan and the network for NGOs is not that strong yet. |
| | | Respondent 5, Female, Teacher Rural, Age 23, Pos. 52 | Interviewee: Like I said before for schools, the only party who can actually make sure that these kind of seminars are conducted is the area education officer. That varies from every district to district. For every district it's a separate AEO. If you're lucky, you get a good AEO who is efficient. They'll put in the effort. Otherwise there's no other way to make sure that this happens. If an AEO is against situations like these, they will not even let… they will make sure that the principals don't let any seminar be conducted in their schools. The AEO does have that kind of power and authority over the principals. Yeah, that's the only approval you need. |
| | | Respondent 5, Female, Teacher Rural, Age 23, Pos. 58 | Interviewee: They don't engage directly but they will engage (AEO)… For example, recently we had an interview with our area education officer. She wanted us to get some NGO on board and have these conversations with our students. She didn't reach out to the NGO herself; she put us to the task. That's usually the way they go. They will reach out to somebody else and they'll want a middle man, because I don't think they want direct involvement with an NGO like that. |

| | | | |
|---|---|---|---|
| | | Respondent 5, Female, Teacher Rural, Age 23, Pos. 69-70 | Yes. If your AEO isn't the problem then your principal probably is. If the principal isn't a problem, these schools have really strong lobbies of teachers. You'll probably have an Islamiat teacher who is willing to put up a fight for everything like this. Even the teachers, because they're born in these communities and they all live in these communities, they carry a lot of the same values that these community members do, so they will also take all of this very personally. If you try to bring about a change in the school and a change in the mentality, they will take it as a personal attack. |
| | | Respondent 5, Female, Teacher Rural, Age 23, Pos. 137-138 | Interviewee: I think one thing that needs to be done is get all area education officers in one place and have somebody influential speak to them. The thing is they don't take NGOs very seriously. When you look at the hierarchy, because they're government officials and NGOs aren't, they think of us as a lot of spoiled kids who are running around thinking we're doing something. That's the way they even speak to us. I think what needs to be done is some health minister and education minister, they need to step up and have a proper, very candid conversation about this. Once the AEOs are on board, then they do have access to funds and they do have that kind of power over their principals so that then they can make them do whatever they want. I think the first point of contact should be AEOs. Even if the principal wants to take an initiative and the AEO is against it and the AEO gets wind of it, the principal is going to have a really hard time surviving. |
| | | Respondent 5, Female, Teacher Rural, Age 23, Pos. 142-143 | Interviewer: Anything that might work for the religious leaders?<br><br>Interviewee: I would like to say yes, but I can't really think of something that will be a sure-shot effective way. That's, I think, a very, very, very uphill battle. An easier battle to fight will be getting in touch with the AEOs and maybe get the government involved with some NGOs and to do something like that. Religious leaders are a very, very tough nut to crack. |
| | | Respondent 11, Female, Teacher Urban, Age 42, Pos. 67 | Interviewee: The support is there in terms of if we talk about -- Again, it's coming from a very small group of people. But how are they going to show their support? Maybe by sharing, because again, if it's coming on the social media, sharing videos, to some extent talking to their own kids. But again, the number is very limited. |
| | | Respondent 11, Female, Teacher Urban, Age 42, Pos. 102 | Again, the same thing, because our culture is and our social norms, they probably are going to come in between and not everybody will be very supportive. Even if the thing that is a need, even they understand the importance, but they're not going to support it in terms of actually participating and supporting maybe those people or maybe joining or coming to this seminar, they might but a lot of people won't. |
| | | Respondent 11, Female, Teacher Urban, Age 42, Pos. 123 | So, I think it's going to take many years, but it has to start from somewhere. Putting stuff on social medias, putting stuff on billboards, printing, having small ads for TV, having small kind of messages that might be set on maybe radio just like we do it for smoking. So, those things have to start in, it must start as a campaign. And if it's done on a smaller scale, it's just going to target maybe a few people. If it's done on a larger scale, and involving the leadership, then it might be effective. |
| | | Respondent 13, Male, Doctor, Age 35, Pos. 17 | However on individual level, the masses on their own and are very active members. They used the social media to upload the images, even in the case of domestic violence, where the underage child labor is being subjected to violence by their owners or the house there, where they are serving. |
| | **5.1. Community support** | Respondent 14, Male, Teacher Rural, Age 23, Pos. 28 | Interviewee: I know that teachers might and a few parents might (be supportive), but the general population might be a bit uncomfortable about it. |
| | | Respondent 25, Male, NGO Employee, Age 29, Pos. 46 | Interviewee: I feel that philanthropists are definitely a tier that I would include in that. They definitely are supportive in this regard. Universities or academic settings, because academic settings do not discriminate on gender. I acknowledge that there are incidents where gender tends to bias decisions as far as professors and other controversies related that is concerned, but because we're focusing only on sexual education and overall continual improvement in that specific field, they are definitely included in this aspect. Academic settings do support sexual education and at least discussing it overall. And lastly, I feel that community health workers, especially women community health workers, they do have a stake in it and they do play their role. They do take these aspects seriously. |
| | | Respondent 28, Female, Health Department, Age 26, Pos. 41 | Interviewee: So I guess in my personal opinion, the support is usually through the like the media, social media, not the electronic or, you know, print media or the social media. If there is a campaign run through the social media, people might, you know, share it, support it, maybe put… show their support through that. Otherwise, if you are talking about the community in general, then at one factor that if they are sensitized about it, then there might be efforts, otherwise then from the political end, if the things are enforced by law or they are made compulsory, or they are just, there are people are subjected towards it to do so then it would be, otherwise not. |
| | | Respondent 1, Female, NGO Employee, Age 27, Pos. 4 | It depends on what kind of schools you want the implementation to be in, because if you see there's a lot of class distribution in Islamabad, there's more of the middle class than the lower class and then the elite class. Now, I don't think that the elite class wouldn't have any problem, because the way their lifestyle is and the way they're living and the way their perceptions are, they're more modernized and westernized. So I don't think that they would have a problem with that. Their parents might have a problem with it being like more of like an open subject, an open discussion. |
| | | Respondent 1, Female, NGO Employee, Age 27, Pos. 5 | But again, they're more adapting to the westernized culture, I would say. Compared to the middle age at the middle income and the lower income classes, I think that their pride is more in their culture and traditions. So if you see that the way how they percept it, it would be very difficult for them to adjust to the fact that their children are learning about sexual education, more openly in schools. And their children may be going to government schools or government colleges, where the ideology of having that kind of subject being opened to the children would be very cumbersome in a sense. |
| | | Respondent 1, Female, NGO Employee, Age 27, Pos. 46-47 | Interviewer: And what about parents or for instance, teachers, would they be supportive or would they not be supportive?<br><br>Interviewee: Again, I would say it would depend on the tier level. So there are private schools, there are public schools and then there are government schools. In government schools, I would say it would be a tough decision to make, because again lower income people are able to afford this. Middle income families also like are able to afford government and public schools. But again, government schools have that ideology, you know, that I've heard that their syllabus hasn't even changed over the years since they've started the curriculum. So kind of changing that perspective about teachers and students there, even their parents, it will be pretty hard. |
| | | Respondent 10, Male, Political Activist, Age 30, Pos. 6 | I would say, one, that a lot of these communities, they are involved in a very day-to-day struggle regarding their survival and their housing and things like that. So, they're not very concerned -- and the reason I didn't rate it 1 was there is a recognition that these things happen and that they know that these things happen but they're not very high on their concern list. For example, the most concerned right now is about their housing rights, whether they have a house or not, or their source of income and stuff like that. So, this is on their list of priorities, for example, and in the daily routine, this is I think, becomes very low in their perceptions. |
| | | Respondent 16, Female, Parent, Age 31, Pos. 42 | There were two things that I myself noticed that either it's not exactly related to the education of the mother. It is not final or he or she needs some education, but there are some mothers who are educated, but they're working and they don't spend time with their children. And they're so busy and occupied in their own life, that they don't want to deal with this issue. They have to take time off with your team. You can't just do it once a month, once a week, you have to do it continuously, time and over and over again. So there are people, who are really busy in the life when they're really told, okay, we saw this happening to you kid, we saw your kid doing this to another kid. So they're in denial! |
| | **5.2. Socio economic status and demographics** | Respondent 17, Male, Parent, Age 29, Pos. 4 | Interviewee: If you look at Islamabad, generally, it's considered to be a much safer city compared to other cities in Pakistan, and generally, we've witnessed fewer cases of gender violence or sexual violence against children in Islamabad. Having said that, it is a concern for the parents because a lot of the residents of Islamabad they're A. Educated, B. They're from working-class. And I would say that they're more open-minded to address these questions and to address this sexual reproductive health with their children. |
| | | Respondent 25, Male, NGO Employee, Age 29, Pos. 22 | Interviewee: Yes, I think there are. As far as I know, I feel that there is a dichotomy. There is basically a dichotomy of responses because those who are working in the cities, those who actually go to the cities in order to gain higher education, amongst goals, I know that they understand these topics because it is discussed in university campus and in academic circles. However, as far as villages are concerned, as far as residents who are of limited education, especially among male workers in villagers, this is a topic which they feel will, and I'm putting this in quotation marks, affect the social standing their women and empowering is often seen as something of a taboo. So, they don't actually promote it amongst the female children that they have. Amongst female siblings overall. |
| | | Respondent 25, Male, NGO Employee, Age 29, Pos. 23 | Interviewer: This is definitely also seen from a socio-economic perspective, because when a family has higher education, if a mother has a better education, and she is able to impart this on her children, they are much more sexually aware, and they're much more educated and they're willing to talk about it more in contrast to those who have limited education and don't have an opportunity, except to survive. |
| | | Respondent 25, Male, NGO Employee, Age 29, Pos. 28 | Because when you have a male-centric establishment when you have a patriarchy, I feel that any topic of discussion on sexual education and by extension women empowerment is impeded. There isn't an effort put in to at least inculcate it in academic circles. And if you don't inculcate it over there, you can't expect it to grow to the public platform overall. It's only educated people who eventually bring the trickle-down effect for this to take this. |
| | | Respondent 4, Female, Teacher Urban, Age 24, Pos. 5 | Interviewee: But I feel like the further away you get from central Islamabad, the worse the problem gets and it's not because of education or access to education, it's more of a culture and mindset. And maybe even access to healthcare, I would say, access to healthcare and access to the authority. |
| | | Respondent 5, Female, Teacher Rural, Age 23, Pos. 29 | Interviewee: Most of them are uneducated completely. They're all daily wagers, the dads and the mothers. The mothers are… Mostly, in our experience we've seen that most of the fathers don't like to earn. They stay at home. They're usually involved in drugs. The mothers, they're sent out to work. They will either go clean houses, cook food, do some tailoring. I think that because their exposure is pretty limited, what they work on is what they've learned, what they've been taught from the villages. They still carry that mentality because nobody has taught them otherwise. Because of that and also because the mothers also work full time and they usually have more than five kids, for the majority, it's very hard for them, I think, to come down to that level and actually spend that kind of time with their children. The mothers who are slightly educated are a bit more open to it but, again, it's just a community mentality that everything like this is a stigma. |
| **5. Dimensions of community support** | | Respondent 1, Female, NGO Employee, Age 27, Pos. 70 | I think community members would show their support by highlighting the problems that have emerged by not having this topic be discussed among the youth. So as I told you, recently, like incidents such as, you know, rape et cetera, have been so common that they can highlight these issues and tell people that, especially girls not knowing what is happening to them and then not knowing anything about this topic, it would be high time to actually highlight this topic among the youth and let them know that this is an emerging problem. |
| | | Respondent 11, Female, Teacher Urban, Age 42, Pos. 59 | They passively support the understand the importance with everybody because they can't take an initiative and they're scared, and then since there's so many myths when it comes to sexual education, I think we need to first resolve those myths before, actually starting to introduce such thing in a curriculum, such kind of. |
| | | Respondent 12, Female, Health Department, Age 36, Pos. 25-26 | Interviewer: Okay. And how do they show their support?<br>Interviewee: A lot of media influencers, a lot of actors and a lot of leaders are part of campaign to advocate it, to make it common. So that's how they support it. Yeah. |
| | **5.3. Passive community support** | Respondent 13, Male, Doctor, Age 35, Pos. 33-34 | Unfortunately the support at Twitter, like I mentioned, I targeted that criteria or that sector of our population that does not use with … they do have a Smartphone, but Twitter is an app, which is mainly restricted. That is the dialogue of our society, that these child laborers are illiterate. They don't know how to use Twitter. So they need to adopt a media, which is comprehendible, even for the illiterate and someone, who's educated. I mean look at the literacy criteria of Pakistan, someone who can just read and write his name and can sign. |
| | | Respondent 13, Male, Doctor, Age 35, Pos. 60 | Passively, yes. Everybody wants to be a part of the bandwagon, on social media, yes, they can read their fingers in write process and long supportive sentence with hashtag this and that, but when they actually get to tag and have to perform that physically and practically. |
| | | Respondent 19, Male, Parent, Age 32, Pos. 36-37 | Interviewee: Generally, it's not a very active support. It's a very passive support. So, they can be talking about them on the Facebook groups or if an incident rises just like these rape cases that have been voiced lately over here in Pakistan. So, one issue is talked about, another issue arises. So, that means that everyone knows about something that is happening around them, their surroundings, or something that is not of use. So, they start raising their voice on those groups, but I don't think it's on a bigger scale. Those groups and these Facebook pages have got their own strength. They can lead to a lot of big things. But it is haphazard, I would say. If it was in a formulated way, it can go much further. |
| | | Respondent 35, Male, NGO Employee, Age 51, Pos. 42 | People show good support to the different things, but behind the scenes, not in front of everybody, behind the scenes. |
| | | Respondent 1, Female, NGO Employee, Age 27, Pos. 11 | Recently if you look at the incidents that have been happening in Islamabad especially all over Pakistan, parents have been more careful with their children. And for example, letting them know, like, you know, as you know good touch and bad touch, stuff like that. That also comes under, I think reproductive health and that kind of you could say part of it, |
| | | Respondent 1, Female, NGO Employee, Age 27, Pos. 27 | Currently… as I told you the incident that have been happening all over the country in Pakistan, publicly on television, they've been contact… like letting people know, bringing out public awareness among parents and children to teach their children about bad touch and good touch. But on that matter, nothing about sexual reproductive health, I would say. |
| | | Respondent 1, Female, NGO Employee, Age 27, Pos. 114 | If it comes in the news, they say, Oh God, you know, it's very bad that that happened, but they don't think why did this happen, you know, what's the root cause analysis of that. They don't think about that. |
| | **5.4. Community** | Respondent 11, Female, Teacher Urban, Age 42, Pos. 11 | Interviewee: Yeah. Because in the past few years, we had some child abuse cases that the case of Zainab and other ones. After that, I think the awareness is there, and they are people who think that this is important, but at the same time, it's considered to be inappropriate to discuss anything related to adolescent health or sex education, Pakistan. So, they just doing it in a very different way, posting videos. And that would include all the age groups. I came across the videos, and they have several slogans for very young girls, particularly. And in terms of for adults, what I've seen is more with the domestic abuse and other kinds of abuse. |

| | | | |
|---|---|---|---|
| | **5.4. Community support post violence** | Respondent 17, Male, Parent, Age 29, Pos. 14 | Interviewee: This particular topic is, I think, very tricky to speak about in Pakistan generally, and leadership, I've seen they generally refrain from commenting on these issues or creating controversy around them. That's something that they completely avoid. So, even in the aftermath of cases like the Zainab incident in Kasur, you had very few politicians that were actually making statements regarding the same issue. So, generally, I would say the fear factor is there, that they are concerned that the clergy and the right-wing people are going to respond not very positively to them campaigning or advocating this. |
| | | Respondent 18, Female, Teacher Urban, Age 30, Pos. 6 | In addition, as a mother, what I observed once I put my daughter in school was that at a playgroup level— because there was the Zainab rape case, very popularized in Pakistan to the point that the Senate even passed a law against child abuse, et cetera— they started introducing the concept of good touch and bad touch. My daughter came home with a pamphlet. The school was sending that to all of the parents, and they wanted the children have this discussion around it. It began from very small, little steps, like when you're going to the bathroom you shouldn't have other class fellows around you just to be able to see you. Little, little things like that. |
| | | Respondent 18, Female, Teacher Urban, Age 30, Pos. 7 | Overall, in Pakistan it is more reactive in nature. I think the stats are staggering. We have like 1 in 10 children or 10 to 15 children abused per day. It's an unfortunate situation. I think what's happening is all of the measures that are being taken are very, very reactive instead of proactive. They revolve more so around prevention of sexual exploitative activities as compared to general health. But, yes, I think it's starting to take place specifically in Islamabad. |
| | | Respondent 23, Female, Parent, Age 29, Pos. 23 | Interviewee: I think it's of a huge importance to the general community in Islamabad or in Pakistan because, like I said, there have been cases now everybody is realizing that this must be taken seriously. So, I think concerns are raised drastically. |
| | **5.5. Changing norms** | Respondent 1, Female, NGO Employee, Age 27, Pos. 19-21 | I think it's a very high priority that it's high time that these things… this topic should be discussed in schools in colleges. The reason is, because that students, who… so the life of a student nowadays what happens is that, especially in Islamabad, like the culture has changed over the years that I've lived in Islamabad for the past 10 years. And the culture is that now the youth especially are more considerate in, for example, getting into relationships, knowing other people before they're getting married. And now the … you could say the style of having an arranged marriage is more overcome by, you know, getting to know the person first and then getting married, for example. So that's … I think that's the image that people are now getting in their kids. And the youth is also getting this image. So I feel like it's high time that this topic is more discussed, so that girls… not especially, not even girls, especially guys get to know like what their sexual reproductive health system is made of and how it can be a part in a relationship, you know. So, I mean, some people may take bad decisions in doing stuff, but actually knowing it before doing something is much better than you don't know anything. So I know that I've seen it in the US in my high school and they usually have a PE class, in which they go over everything related to the sexual reproductive health system, the organs everything. So people know what they're actually doing compared to not what they're doing and then trying it. So it's more of like diving in and knowing what you're doing. |
| | | Respondent 1, Female, NGO Employee, Age 27, Pos. 74 | So by… opposing means that they might not agree to such topics being highlighted openly. So like right now in Pakistan, even though it's adapting more to towards modernism, such topics are still not being discussed in the community. And there's a reason for that, that how are you supposed to explain such, you know, emerging topics to your kids? So I mean, it would be… parents would be opposing this, like why such topics are being openly discussed. And kids coming to them and asking them questions, you know that today I saw this, or I read this, so what does this mean? So how would parents be able to explain in the naive way that, you know, this means this, and that means that. |
| | | Respondent 29, Female, Head of School, Age 29, Pos. 30-31 | Interviewee: Because personally I've seen just a lot of children and general young adults engaging in things that they could have easily avoided had they gotten the right information, even the youngest of children. I mean, I work with preschoolers and I've seen so much sexual abuse and people being in so much denial about it, but there is domestic staff and all of that. And even with young adults just experimenting with their sexualities, had they gotten the right information, maybe or if this was an open dialogue for them, there was just a lot that could have been avoided. And this is some kind of like, obviously I know that this is a trauma that they carry for the rest of her life and it's a huge deal. So it's a very, very big concern. And not just from my perspective, but from the people that I talk to on a daily basis, it's like a very hotly debated topic that why is this not still being addressed? So, yeah. |
| | | Respondent 34, Female, Social Media Influencer, Age 38, Pos. 45-46 | Interviewee: Because I still think that people from my generation are more concerned about it. They're more concerned about the sexual education for their kids, but the previous generation, like our parents were not very concerned about it. That's why we never had that education, no one talked to us about it. No one in school or in family or in any other educational Institute, discuss these things with us. But I think the next generation and because of the… because of the social media and how things are more accessible, we do want our kids to be people in Pakistan, do want their kids to be… to know more about this stuff. So they, when there's everything on the internet, it's very important, I think for them to know what's right and what's wrong. Yeah. |

Supplementary Table 4: For all 35 interviews, descriptive statistics of qualitative coded segments presented below. For each code, mean values are the average coded segments for 35 interviews. The total entries for each code reflect the overall number of coded segments.

| S. No. | Code | Mean | Std. dev.[1] | Min.[2] | Max.[3] | Total entries |
|---|---|---|---|---|---|---|
| 1 | Financial Resources | 4.54 | 2.20 | 1 | 9 | 159 |
| 2 | Information Availability | 1.31 | 1.02 | 0 | 5 | 46 |
| 3 | Experts | 0.97 | 1.10 | 0 | 4 | 34 |
| 4 | Lack of Awareness and Priority | 3.14 | 2.45 | 0 | 9 | 110 |
| 5 | Resistance from Conservatives | 4.11 | 2.14 | 1 | 10 | 144 |
| 6 | Taboo and Silence | 3.17 | 2.47 | 0 | 11 | 111 |
| 7 | Misconceptions | 2.29 | 1.90 | 0 | 7 | 80 |
| 8 | Dangerous consequences | 0.40 | 0.74 | 0 | 3 | 14 |
| 9 | Dissemination of Efforts | 0.37 | 0.91 | 0 | 4 | 13 |
| 10 | Disconnect with the community | 0.29 | 0.79 | 0 | 4 | 10 |
| 11 | Western concept | 0.34 | 0.64 | 0 | 2 | 12 |
| 12 | Strategies for implementation | 1.97 | 1.92 | 0 | 7 | 69 |
| 13 | NGOs | 3.11 | 2.61 | 0 | 11 | 109 |
| 14 | Digital Media social media Online Platforms | 2.57 | 1.75 | 0 | 6 | 90 |
| 15 | Community Engagement | 1.57 | 1.74 | 0 | 6 | 55 |
| 16 | Sexual Violence | 1.29 | 1.76 | 0 | 7 | 45 |
| 17 | Opportunities for Awareness | 1.34 | 1.37 | 0 | 5 | 47 |
| 18 | Age Appropriateness | 0.71 | 0.83 | 0 | 3 | 25 |
| 19 | Masking/Optimizing the CSE | 0.34 | 0.94 | 0 | 4 | 12 |
| 20 | Peer to Peer or Parent to child | 0.14 | 0.43 | 0 | 2 | 5 |
| 21 | PSHE | 0.23 | 1.03 | 0 | 6 | 8 |
| 22 | Leadership | 4.31 | 2.36 | 0 | 12 | 151 |
| 23 | Current Efforts | 2.54 | 1.79 | 0 | 7 | 89 |
| 24 | Government Support | 2.29 | 2.27 | 0 | 11 | 80 |
| 25 | School Heads and Teachers | 2.20 | 2.91 | 0 | 12 | 77 |
| 26 | Policy | 1.40 | 1.61 | 0 | 8 | 49 |
| 27 | Bureaucracy | 0.91 | 1.44 | 0 | 6 | 32 |
| 28 | Community Support | 1.46 | 1.48 | 0 | 6 | 51 |
| 29 | Socio economic status and demographics | 1.86 | 1.72 | 0 | 6 | 65 |
| 30 | Passive Community Support | 1.17 | 1.25 | 0 | 4 | 41 |
| 31 | Community Support Post Violence | 0.69 | 1.18 | 0 | 5 | 24 |
| 32 | Changing Norms | 0.51 | 0.89 | 0 | 4 | 18 |

[1] Standard deviation.
[2] Minimum.
[3] Maximum.

Supplementary Table 5: Inter-code relationship descriptive statistics are presented below. The average linkages between each code and the other 31 codes are represented by mean values. The total number of linkages between a code and the other 31 codes is represented by total linkages.

| S. No. | Code | Mean | Std. dev. | Min. | Max. | Total linkages |
|---|---|---|---|---|---|---|
| 1 | Financial Resources | 4.55 | 7.42 | 0 | 32 | 141 |
| 2 | Information Availability | 1.97 | 2.64 | 0 | 12 | 61 |
| 3 | Experts | 0.71 | 1.35 | 0 | 6 | 22 |
| 4 | Lack of Awareness and Priority | 4.52 | 5.22 | 0 | 19 | 140 |
| 5 | Resistance from Conservatives | 5.81 | 7.94 | 0 | 32 | 180 |
| 6 | Taboo and Silence | 5.42 | 8.14 | 0 | 31 | 168 |
| 7 | Misconceptions | 3.48 | 6.40 | 0 | 29 | 108 |
| 8 | Dangerous consequences | 0.81 | 2.02 | 0 | 11 | 25 |
| 9 | Dissemination of Efforts | 0.42 | 0.76 | 0 | 3 | 13 |
| 10 | Disconnect with the community | 0.52 | 0.77 | 0 | 3 | 16 |
| 11 | Western concept | 0.55 | 1.39 | 0 | 6 | 17 |
| 12 | Strategies for implementation | 3.32 | 4.48 | 0 | 19 | 103 |
| 13 | NGOs | 4.90 | 7.99 | 0 | 32 | 152 |
| 14 | Digital Media social media Online Platforms | 3.74 | 3.75 | 0 | 12 | 116 |
| 15 | Community Engagement | 2.94 | 4.39 | 0 | 19 | 91 |
| 16 | Sexual Violence | 2.48 | 3.17 | 0 | 12 | 77 |
| 17 | Opportunities for Awareness | 2.42 | 3.60 | 0 | 14 | 75 |
| 18 | Age Appropriateness | 0.87 | 1.93 | 0 | 9 | 27 |
| 19 | Masking/Optimizing the CSE | 0.55 | 1.03 | 0 | 4 | 17 |
| 20 | Peer to Peer or Parent to child | 0.19 | 0.48 | 0 | 2 | 6 |
| 21 | PSHE | 0.32 | 0.54 | 0 | 2 | 10 |
| 22 | Leadership | 6.58 | 8.66 | 0 | 32 | 204 |
| 23 | Current Efforts | 3.84 | 6.18 | 0 | 26 | 119 |
| 24 | Government Support | 4.87 | 7.90 | 0 | 30 | 151 |
| 25 | School Heads and Teachers | 3.84 | 4.11 | 0 | 17 | 119 |
| 26 | Policy | 1.52 | 1.90 | 0 | 9 | 47 |
| 27 | Bureaucracy | 1.87 | 4.10 | 0 | 17 | 58 |
| 28 | Community Support | 2.42 | 2.80 | 0 | 10 | 75 |
| 29 | Socio economic status and demographics | 2.61 | 3.82 | 0 | 17 | 81 |
| 30 | Passive Community Support | 1.87 | 2.74 | 0 | 12 | 58 |
| 31 | Community Support Post Violence | 1.03 | 1.40 | 0 | 6 | 32 |
| 32 | Changing Norms | 1.00 | 1.57 | 0 | 6 | 31 |

Supplementary Table 6: The absolute values of Pearson correlation coefficients computed for inter-code relationships are provided as a correlation matrix. The first row's numbers (1-32) correspond to the first column's code serial number (e.g., 1=Financial Resources, 2=Information Availability, etc.). The correlations between each code and itself are represented by the diagonal element (in red), that's why they are always one. A steric (*) is used to indicate correlations that are significant at the 1% level. Only correlations that were significant at 1% were considered in the network analysis.

| S. No. | Codes | 1 | 2 | 3 | 4 | 5 | 6 | 7 | 8 | 9 | 10 | 11 | 12 | 13 | 14 | 15 | 16 | 17 | 18 | 19 | 20 | 21 | 22 | 23 | 24 | 25 | 26 | 27 | 28 | 29 | 30 | 31 | 32 |
|---|---|---|---|---|---|---|---|---|---|---|---|---|---|---|---|---|---|---|---|---|---|---|---|---|---|---|---|---|---|---|---|---|---|
| 1 | Financial Resources | 1.00 | 0.35 | 0.6* | 0.14 | 0.26 | 0.09 | -0.02 | 0.10 | 0.64* | 0.25 | -0.13 | -0.07 | 0.57* | 0.11 | 0.16 | 0.68* | -0.11 | -0.06 | 0.01 | -0.09 | 0.02 | 0.56* | 0.8* | 0.75* | 0.18 | 0.54* | 0.79* | 0.35 | 0.12 | 0.14 | 0.31 | 0.06 |
| 2 | Information Availability | | 1.00 | 0.52* | 0.06 | -0.05 | 0.13 | 0.15 | 0.03 | 0.25 | 0.09 | -0.12 | 0.16 | 0.13 | -0.14 | 0.01 | 0.64* | 0.15 | 0.15 | -0.07 | 0.10 | 0.29 | 0.22 | 0.39 | 0.15 | 0.14 | 0.00 | 0.07 | 0.24 | 0.24 | 0.46 | 0.09 | 0.58* |
| 3 | Experts | | | 1.00 | 0.00 | -0.21 | -0.03 | -0.13 | -0.08 | 0.44 | 0.17 | -0.21 | -0.05 | 0.35 | 0.02 | -0.02 | 0.46 | -0.03 | -0.08 | 0.09 | -0.02 | 0.32 | 0.23 | 0.61* | 0.32 | -0.02 | 0.10 | 0.19 | 0.07 | 0.15 | 0.00 | 0.02 | -0.03 |
| 4 | Lack of Awareness and Priority | | | | 1.00 | 0.59* | 0.67* | 0.56* | 0.24 | -0.01 | 0.41 | 0.37 | 0.04 | 0.14 | 0.16 | 0.12 | 0.09 | 0.02 | 0.47* | -0.03 | 0.31 | 0.08 | 0.22 | 0.15 | 0.05 | 0.6* | 0.12 | 0.17 | 0.15 | 0.81* | 0.20 | 0.04 | 0.31 |
| 5 | Resistance from Conservatives | | | | | 1.00 | 0.55* | 0.56* | 0.63* | 0.09 | 0.40 | 0.39 | 0.01 | 0.04 | 0.26 | 0.38 | 0.12 | 0.11 | 0.5* | -0.06 | 0.12 | -0.04 | 0.08 | -0.02 | 0.26 | 0.58* | 0.17 | 0.28 | 0.32 | 0.36 | 0.28 | 0.11 | 0.18 |
| 6 | Taboo and Silence | | | | | | 1.00 | 0.79* | 0.73* | 0.28 | 0.62* | 0.77* | 0.25 | -0.09 | 0.22 | 0.14 | 0.10 | 0.21 | 0.65* | 0.32 | 0.42 | 0.05 | 0.34 | 0.05 | -0.01 | 0.37 | -0.04 | 0.07 | 0.26 | 0.67* | 0.01 | 0.06 | 0.16 |
| 7 | Misconceptions | | | | | | | 1.00 | 0.48* | 0.01 | 0.40 | 0.59* | 0.09 | -0.25 | 0.03 | 0.15 | 0.10 | 0.17 | 0.74* | 0.17 | 0.11 | -0.19 | 0.30 | -0.11 | -0.25 | 0.55* | -0.18 | -0.15 | 0.00 | 0.63* | 0.09 | -0.19 | 0.42 |
| 8 | Dangerous consequences | | | | | | | | 1.00 | 0.48* | 0.30 | 0.74* | 0.30 | -0.05 | 0.22 | 0.14 | 0.03 | 0.25 | 0.19 | 0.56* | -0.03 | -0.04 | 0.53* | 0.08 | 0.04 | 0.07 | 0.02 | 0.09 | 0.12 | 0.17 | -0.01 | 0.12 | -0.05 |
| 9 | Dissemination of Efforts | | | | | | | | | 1.00 | 0.18 | 0.31 | 0.09 | 0.34 | 0.32 | 0.14 | 0.61* | 0.17 | 0.03 | 0.46 | -0.15 | 0.14 | 0.40 | 0.61* | 0.49* | 0.18 | 0.34 | 0.43 | 0.12 | -0.02 | -0.15 | 0.13 | -0.18 |
| 10 | Disconnect with the community | | | | | | | | | | 1.00 | 0.35 | 0.21 | -0.06 | 0.00 | 0.10 | 0.14 | 0.24 | 0.56* | 0.17 | 0.26 | -0.11 | 0.23 | 0.03 | 0.05 | 0.35 | -0.21 | 0.00 | 0.34 | 0.63* | 0.00 | -0.19 | 0.10 |
| 11 | Western concept | | | | | | | | | | | 1.00 | 0.04 | -0.24 | 0.19 | 0.11 | -0.19 | 0.14 | 0.65* | 0.29 | 0.18 | 0.06 | 0.18 | -0.12 | -0.21 | 0.09 | -0.12 | -0.12 | -0.19 | 0.20 | -0.13 | -0.11 | -0.02 |
| 12 | Strategies for implementation | | | | | | | | | | | | 1.00 | -0.04 | 0.24 | 0.66* | -0.01 | 0.9* | -0.06 | 0.67* | 0.27 | 0.05 | 0.26 | 0.02 | -0.16 | 0.04 | 0.17 | 0.02 | 0.55* | 0.00 | 0.18 | 0.10 | 0.23 |
| 13 | NGOs | | | | | | | | | | | | | 1.00 | -0.02 | -0.16 | 0.45 | -0.20 | -0.24 | -0.18 | -0.20 | 0.43 | 0.53* | 0.66* | 0.75* | 0.14 | 0.52* | 0.53* | 0.18 | -0.06 | 0.14 | 0.29 | -0.17 |
| 14 | Digital Media Social media Online | | | | | | | | | | | | | | 1.00 | 0.5* | 0.11 | 0.35 | 0.14 | 0.51* | 0.09 | 0.09 | 0.04 | -0.02 | 0.08 | 0.27 | 0.26 | 0.06 | 0.5* | 0.08 | 0.28 | 0.58* | 0.16 |
| 15 | Community Engagement | | | | | | | | | | | | | | | 1.00 | 0.06 | 0.8* | 0.18 | 0.55* | 0.35 | 0.05 | 0.04 | -0.02 | 0.01 | 0.44 | 0.31 | 0.03 | 0.36 | 0.11 | 0.23 | 0.17 | 0.39 |
| 16 | Sexual Violence | | | | | | | | | | | | | | | | 1.00 | -0.05 | 0.01 | -0.02 | 0.10 | 0.05 | 0.32 | 0.7* | 0.58* | 0.45 | 0.30 | 0.43 | 0.31 | 0.26 | 0.19 | 0.24 | 0.23 |
| 17 | Opportunities for Awareness | | | | | | | | | | | | | | | | | 1.00 | 0.12 | 0.69* | 0.01 | 0.10 | 0.12 | -0.09 | -0.20 | 0.24 | 0.06 | -0.20 | 0.41 | 0.13 | 0.26 | 0.06 | 0.37 |
| 18 | Age Appropriateness | | | | | | | | | | | | | | | | | | 1.00 | -0.09 | 0.42 | 0.13 | -0.05 | -0.19 | -0.21 | 0.41 | -0.19 | -0.19 | -0.02 | 0.59* | 0.09 | -0.22 | 0.33 |
| 19 | Masking Optimizing the CSE | | | | | | | | | | | | | | | | | | | 1.00 | 0.11 | -0.04 | 0.27 | 0.01 | -0.12 | 0.14 | 0.15 | -0.09 | 0.23 | 0.03 | -0.15 | 0.07 | -0.01 |
| 20 | Peer to Peer or Parent to child | | | | | | | | | | | | | | | | | | | | 1.00 | 0.00 | -0.18 | -0.10 | -0.22 | 0.31 | -0.01 | -0.11 | 0.10 | 0.38 | -0.14 | -0.17 | 0.22 |
| 21 | PSHE | | | | | | | | | | | | | | | | | | | | | 1.00 | 0.08 | 0.12 | 0.05 | 0.02 | 0.18 | 0.12 | -0.02 | 0.02 | 0.13 | 0.10 | 0.07 |
| 22 | Leadership | | | | | | | | | | | | | | | | | | | | | | 1.00 | 0.55* | 0.51* | 0.16 | 0.55* | 0.64* | 0.28 | 0.20 | 0.27 | 0.29 | 0.17 |
| 23 | Current Efforts | | | | | | | | | | | | | | | | | | | | | | | 1.00 | 0.59* | -0.06 | 0.6* | 0.73* | 0.10 | 0.06 | 0.13 | 0.29 | -0.04 |
| 24 | Government Support | | | | | | | | | | | | | | | | | | | | | | | | 1.00 | 0.21 | 0.59* | 0.84* | 0.35 | -0.07 | 0.13 | 0.37 | -0.20 |
| 25 | School Heads and Teachers | | | | | | | | | | | | | | | | | | | | | | | | | 1.00 | 0.11 | 0.00 | 0.23 | 0.65* | 0.10 | -0.05 | 0.41 |
| 26 | Policy | | | | | | | | | | | | | | | | | | | | | | | | | | 1.00 | 0.79* | 0.22 | -0.10 | 0.23 | 0.39 | 0.11 |
| 27 | Bureaucracy | | | | | | | | | | | | | | | | | | | | | | | | | | | 1.00 | 0.34 | -0.03 | 0.15 | 0.44 | -0.05 |
| 28 | Community Support | | | | | | | | | | | | | | | | | | | | | | | | | | | | 1.00 | 0.19 | 0.47* | 0.59* | 0.30 |
| 29 | Socio economic status and demographics | | | | | | | | | | | | | | | | | | | | | | | | | | | | | 1.00 | 0.08 | -0.10 | 0.40 |
| 30 | Passive Community Support | | | | | | | | | | | | | | | | | | | | | | | | | | | | | | 1.00 | 0.45 | 0.6* |
| 31 | Community Support Post Violence | | | | | | | | | | | | | | | | | | | | | | | | | | | | | | | 1.00 | 0.06 |
| 32 | Changing Norms | | | | | | | | | | | | | | | | | | | | | | | | | | | | | | | | 1.00 |
